# Decoding Structure‐Property Relationships in Anion Exchange Membranes via a Chemically Informed Dual‐Channel Graph Attention Network

**DOI:** 10.1002/advs.74971

**Published:** 2026-04-02

**Authors:** Wanting Chen, Ye Hu, Zijun Xiao, Deming Xia, Bo Pang, Xuemei Wu, Gaohong He

**Affiliations:** ^1^ State Key Laboratory of Fine Chemicals Frontier Science Center For Smart Materials School of Chemical Engineering Dalian University of Technology Dalian China; ^2^ Key Laboratory of Industrial Ecology and Environmental Engineering (MOE) Dalian Key Laboratory On Chemicals Risk Control and Pollution Prevention Technology School of Environmental Science and Technology Dalian University of Technology Dalian China

**Keywords:** alkaline stability, anion exchange membranes, chemical prior knowledge, machine learning, structure‐property relationship

## Abstract

Anion exchange membranes (AEMs) are key components in emerging energy technologies, yet their development is hindered by the challenge of simultaneously achieving high hydroxide conductivity and durable alkaline stability. These properties are governed by multiscale, coupled effects of polymer architecture, microphase separation, and operating conditions, making AEM exploration slow and largely empirical. Here, we propose SPARK, a structure‐property graph attention network with prior knowledge of chemistry embedding, to accelerate AEM molecular design through robust candidate prioritization and mechanism‐relevant interpretability. SPARK embeds chemical prior knowledge into molecular graphs and employs a dual‐channel architecture that separately encodes hydrophilic ionic and hydrophobic non‐ionic segments, explicitly capturing microphase separation and ion‐transport channel formation. It translates AEM structures into five‐level performance grades for hydroxide conductivity and alkaline stability with high accuracy, outperforming conventional machine‐learning baselines. Attention‐based interpretation further pinpoints structural units associated with ion transport and alkaline degradation, providing actionable guidance to mitigate the conductivity‐stability trade‐off. Finally, SPARK is validated by pre‐grading to‐be‐synthesized AEM candidates with good agreement to experiments, and the accompanying software package is publicly released to facilitate broader adoption and data‐driven AEM design.

## Graphical abstract

SPARK decodes structure‐property relationships in anion exchange membranes (AEMs) via a chemically informed dual‐channel graph attention network (DEGAT) that explicitly captures microphase separation. It outputs five‐level grades for hydroxide conductivity and alkaline stability and highlights relevant key structural units, enabling robust pre‐screening and experimental validation of new AEM candidates.

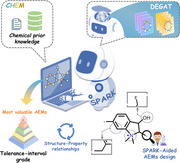

## 1 Introduction

Anion exchange membranes (AEMs) are key components in emerging energy technologies, including water electrolysis, CO_2_ electrolyzers, and fuel cells [1, 2]. Despite thousands of studies over the past decade, their broad application still faces a persistent materials‐design challenge: simultaneously achieving high hydroxide conductivity and durable alkaline stability [3, 4, 5]. These properties arise from coupled effects of molecular‐scale chemical architecture (e.g., cationic groups, polymer backbones, and linkage motifs), mesoscale microstructure (e.g., microphase separation), and operating conditions (e.g., temperature and humidity) [6]. Such multiscale, condition‐dependent relationships render the rational design of AEMs highly challenging [7]. Consequently, conventional trial‐and‐error development remains inefficient, costly, and time‐consuming, severely limiting the exploration of chemical space and potentially overlooking promising candidates [8, 9, 10].

With the rapid development of artificial intelligence, machine learning (ML) provides a data‐driven route to connect AEM polymer structures with key properties. Most ML studies in AEM research rely on continuous‐value regression of ionic conductivity or alkaline stability [11, 12, 13, 14]. However, these experimental measurements often show substantial variability due to differences in membrane fabrication, operating conditions, and testing protocols, resulting in fluctuations of ±10% or more even for nominally identical materials [15, 16, 17]. Such measurement noise, together with training stochasticity, can compromise regression reliability and practical generalization. In addition, AEM datasets are often strongly imbalanced, with relatively few high‐performance samples, making regression prone to biased fitting and overfitting [18].

A more robust alternative is property‐grade classification, in which target properties are discretized into tolerance intervals (grades), thereby weakening the impact of experimental variability and improving predictive stability. This grade‐based strategy has outperformed regression and supported experimental validation in 2D organic metal chalcogenides [19], and has been used to address noise‐limited prediction in polymer thermal conductivity and quantum materials [20, 21]. Practically, grade‐based classification can also serve as a rapid, low‐cost pre‐screening step to deprioritize weak candidates and avoid unnecessary downstream computation [22]. This capability is well suited for future closed‐loop AEM design workflows, where generative models can produce large numbers of chemically plausible candidates under physicochemical constraints, and efforts must be focused on the most promising designs. Moreover, grade‐based outputs align naturally with engineering workflows (e.g., tiered decision‐making or pass/fail validation) and facilitate more interpretable identification of key structure‐property descriptors [23]. Despite these advantages, ML models tailored for property‐grade prediction in AEMs remain largely unexplored.

Current ML models for AEMs face a key bottleneck: extracting transferable, mechanism‐relevant features from chemically complex polymer architectures [24]. Conventional feature engineering, which mainly relies on expert‐crafted descriptors and molecular simulations, is labor‐intensive and often incomplete [25, 26]. This challenge is further amplified for polymers relative to small molecules, as high molecular weight, conformational heterogeneity, and hierarchical microstructures complicate accurate digital representation [27, 28, 29, 30]. Graph attention networks (GATs) provide a compelling alternative by learning molecular representations directly from graphs and adaptively weighting atomic interactions, thereby capturing structure‐property relationships governed by molecular topology and functional groups [31, 32, 33, 34, 35, 36, 37, 38]. However, generic graph encodings may overlook AEM‐specific physics, particularly the hydrophilic‐hydrophobic microphase separation that governs percolated ion‐transport pathways [39]. This limitation highlights two complementary needs for AEMs‐learning models: first, incorporating fundamental chemical prior knowledge (e.g., charge/electronic effects, and hydrogen‐bonding propensity) to endow molecular graphs with richer chemical meaning [40]; second, introducing structure‐aware representations that can disentangle hydrophilic and hydrophobic contributions, because microphase separation is intrinsically a biphasic organization rather than a local graph property. This design anchors representation learning in chemically meaningful cues while enforcing mechanism‐relevant feature disentanglement, thereby improving data efficiency, cross‐condition generalization, and interpretability.

Another underemphasized challenge in ML for AEMs is the effective integration of multi‐scale information, including molecular structures, physicochemical parameters, and experimental conditions. Most existing approaches simply concatenate these inputs or process them in parallel, neglecting their intrinsic couplings [11, 12, 13, 14]. As a result, models may overemphasize non‐structural variables (e.g., temperature) while underutilizing molecular semantics, ultimately degrading generalization. To address this limitation, feature‐wise linear modulation (FiLM), originally developed for visual reasoning [41], can be adapted to condition intermediate neural representations on external variables. In this way, operating conditions dynamically modulate learned molecular features, enabling flexible and sample‐efficient multi‐modal learning.

**FIGURE 1:**
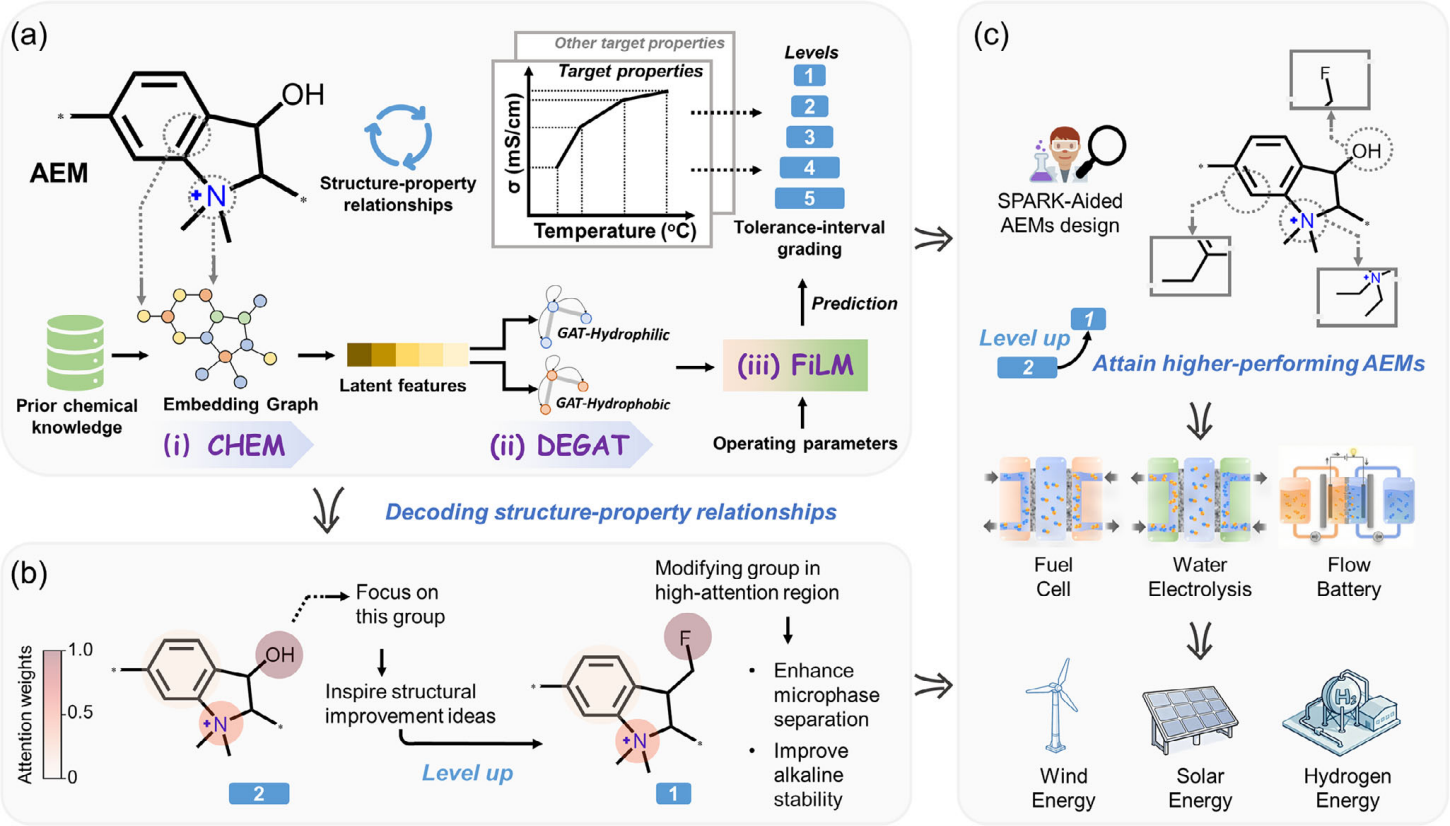
Schematic workflow of the SPARK framework. (a) Sequential integration of prior chemical knowledge embedding (CHEM), a dual‐channel edge‐enhanced graph attention network (DEGAT), and condition‐aware feature‐wise linear modulation (FiLM) for identifying high‐performance AEMs; (b) Visualization of GAT attention weights to enable interpretable AEM structural analysis and design; (c) SPARK‐guided refinement and design of AEM chemical structures for electrochemical applications.

Herein, a structure‐property graph attention network with knowledge embedding (SPARK, Figure 1) is proposed to decode AEM properties from molecular structure and enable robust property‐grade prediction. Prior knowledge of chemistry is first embedded to construct semantically enriched molecular graphs. Motivated by the hydrophilic‐hydrophobic microphase separation, a dual‐channel edge‐enhanced graph attention network (DEGAT) is introduced to separately encode ionic (hydrophilic) and non‐ionic (hydrophobic) segments. In addition, the FiLM module incorporates operating conditions as contextual signals to dynamically regulate intermediate molecular representations. This hierarchical design enables accurate AEM grade prediction, achieving balanced accuracies of 0.8797 for conductivity grading and 0.9152 for stability grading, and outperforming conventional machine‐learning baselines. Attention‐based interpretation pinpoints structural regions associated with ion transport and alkaline degradation, providing actionable guidance to balance conductivity and stability. SPARK is also validated by pre‐grading to‐be‐synthesized AEM candidates with good agreement to experiments, and the software is publicly released to support community adoption and data‐driven AEM design.

## 2 Results and Discussion

### 2.1 Model Overview

**FIGURE 2:**
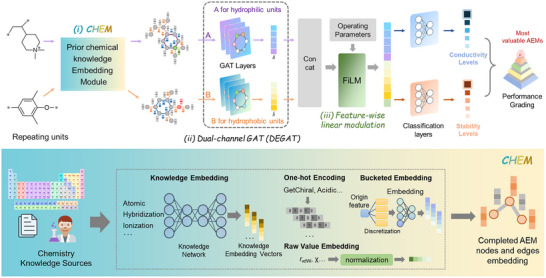
Overview of the SPARK framework. CHEM embeds prior chemical knowledge into molecular graphs; dual channels (DEGAT‐A/B) encode hydrophilic and hydrophobic repeating units; and FiLM integrates operating conditions. These modules operate sequentially to enable five‐level property‐grade prediction and AEM candidate prioritization. “Concat” denotes concatenation along the feature dimension.

SPARK takes simplified molecular input line entry system (SMILES), ion‐exchange capacity (IEC), and operating conditions as inputs, processes them through three sequential feature‐learning modules, and outputs a five‐level performance grade, thereby capturing the underlying structure–property‐condition relationships (Figure 2). As the perceptual front‐end, the prior chemical knowledge embedding module (CHEM) converts SMILES into chemically informed molecular graphs and produces task‐adaptive representations grounded in chemical semantics. CHEM employs four complementary embedding schemes, namely, pretrained, one hot, raw value, and binned embeddings (Figure 2; Table S1). These schemes are tailored to encode high cardinality discrete attributes (e.g., atom and bond types), low cardinality categorical variables (e.g., formal charge and stereochemistry), continuous scalar descriptors, and threshold sensitive hybrid features (e.g., hydrogen bond counts), respectively.

The second module is a dual‐channel edge‐enhanced graph attention network (DEGAT; Figure 2; Figure S1), comprising two dedicated channels (DEGAT‐A/B) that separately encode ionic and non‐ionic repeating units to explicitly represent hydrophilic‐hydrophobic microphase separation. For AEMs with three or more repeating units, the principal hydrophilic and hydrophobic segments are encoded first, followed by incorporation of the remaining units to refine the representation (Figure S2). Self‐supervised feature‐reconstruction pretraining is then used to initialize the encoder weights (Table S2), allowing DEGAT to capture polymer‐relevant structural features, including topological connectivity and interaction effects.

The third module is a multiscale fusion module that incorporates operating conditions into the hydrophilic/hydrophobic embeddings through FiLM. For stability grading, immersion time, temperature, and alkaline concentration are used as conditioning variables, whereas conductivity grading uses temperature only. These variables dynamically modulate intermediate structural representations, enabling adaptation to environmental variations while preserving primary sensitivity to chemical structures. The conditioned representation is finally passed to a fully connected classifier for grade prediction, and the whole network, including the pretrained DEGAT encoders, is fine‐tuned end‐to‐end for robust and interpretable multitask inference.

### 2.2 Data Organization and Statistical Analysis

**FIGURE 3:**
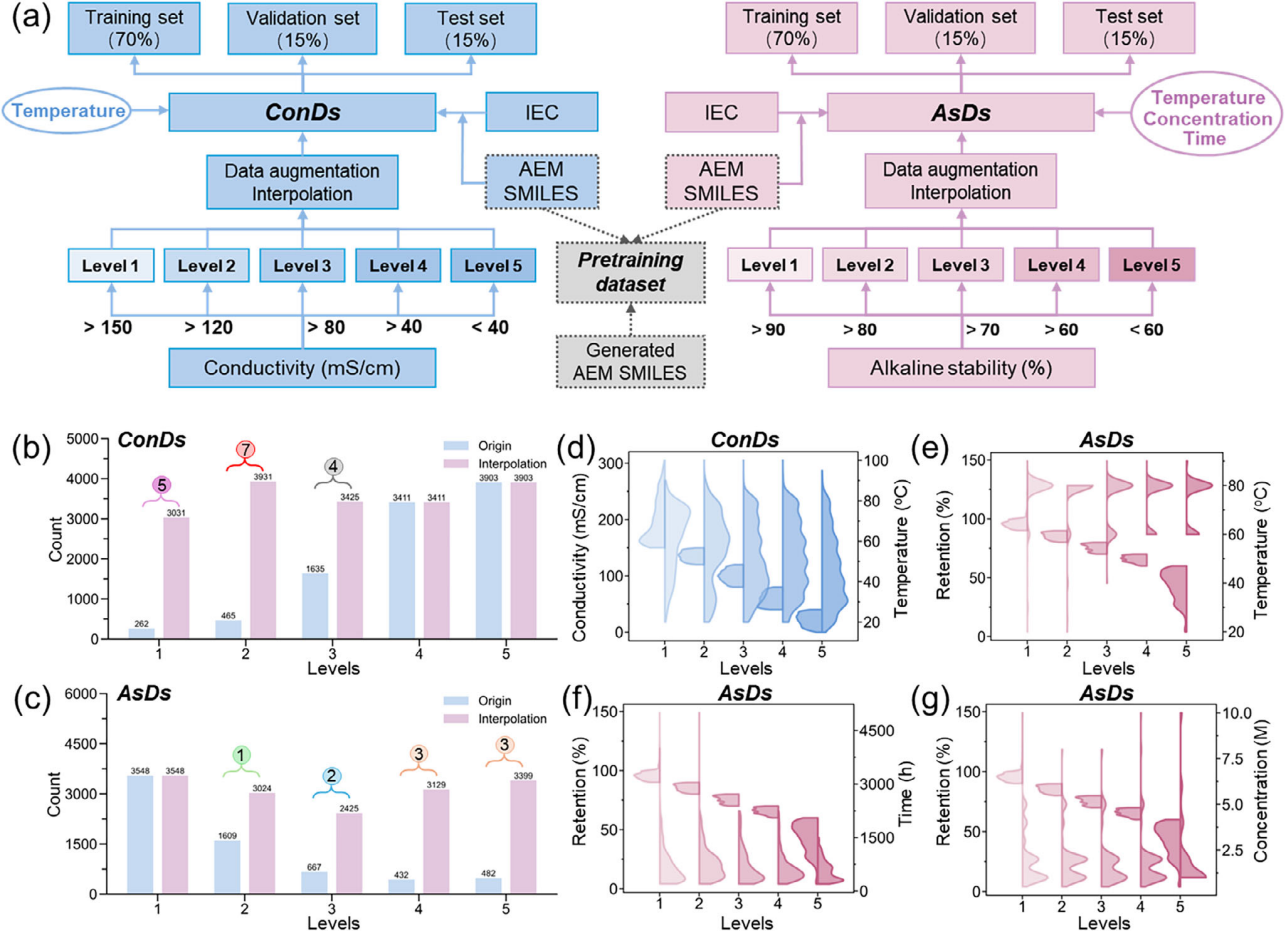
Data collection and statistical analysis. (a) composition of datasets for DEGAT pretraining and model fine‐tuning (ConDs and AsDs); (b, c) grade distributions in ConDs and AsDs before and after data augmentation (numbers in circles denote interpolation counts); (d) hydroxide conductivity versus test temperature across grades in ConDs; (e–g) conductivity or IEC retention under varying test conditions (temperature, immersion time, alkaline concentration) across stability grades in AsDs.

A comprehensive dataset was established to support both self‐supervised pretraining of the DEGAT network and supervised fine‐tuning for property‐grade prediction (Figure 3a). The dataset combines a previously established AEM database [42] with an expanded literature survey to June 2025, yielding 1 320 unique AEM structures with hydroxide‐conductivity and alkaline‐stability data under diverse conditions. For DEGAT pretraining, 22 356 unlabeled structures, including literature‐reported and AI‐generated AEMs in our previous work [42], were used. Generated structures are screened for both chemical validity and synthesizability before being integrated with the experimental set (Table S3). The pretraining set comprises 19 063 hydrophilic ionic repeating units (540 from the literature and 18 523 AI‐generated) and 3 293 hydrophobic non‐ionic repeating units (141 from the literature and 3 152 AI‐generated). These two subsets were separately fed into the corresponding channels to learn segment‐specific structural representations.

A labeled dataset was extracted for fine‐tuning the property‐grading model, containing SMILES, IECs, testing conditions, and grade labels. The dataset was organized into a Conductivity Dataset (ConDs) and an Alkaline Stability Dataset (AsDs). Continuous values of hydroxide conductivity and alkaline stability were discretized into five grades. For conductivity (mS/cm), Levels 5–1 corresponded to (0, 40], (40, 80], (80, 120], (120, 150], (150, +∞), with the highest grade benchmarked against the commercial PiperION membrane. Alkaline stability was quantified by retention metrics (conductivity or IEC retention, %) after alkaline treatment, and Grades 5–1 correspond to (0, 60], (60, 70], (70, 80], (80, 90], (90, 100]. To alleviate class imbalance, data augmentation by linear interpolation between adjacent samples was used (Figure 3b,c) based on near‐linear trends in the source data.

The density distribution of hydroxide conductivity versus test temperature in ConDs (Figure 3d) shows a clear positive correlation between conductivity grade and temperature. Level‐1 samples are enriched near 80°C, consistent with the thermally activated nature of hydroxide transport in AEMs, whereas lower‐grade samples are distributed over a broader temperature range, indicating greater structural and operational variability. Importantly, elevated temperature alone does not ensure high conductivity, underscoring the dominant role of chemical structure. This finding supports the need for an explicit feature‐fusion strategy that preserves structural sensitivity while incorporating operating conditions. The distribution of conductivity/IEC retention across alkaline stability grades under diverse test conditions in AsDs is shown in Figure 3e–g. Most measurements were performed at 80°C (with some at 60°C) in 1–2 M alkaline solutions, whereas some Level‐1 samples still maintained high retention in nearly 5 M alkali, indicating excellent robustness. To improve data reliability, measurements with immersion times below 200 h were excluded. Longer immersion times were more often reported for higher‐grade AEMs, mainly because more stable membranes are typically subjected to extended and harsher tests.

### 2.3 Model Performance

Guided by the widely accepted view that hydrophilic‐hydrophobic microphase separation in AEMs governs the formation and connectivity of ionic channels, the two branches of DEGAT architecture were designed and pretrained separately using hydrophilic and hydrophobic repeating units. During pretraining, feature‐reconstruction performance was monitored. The loss curves converge after about 300 and 200 epochs, respectively (Figures S3–S6), indicating effective feature learning. The pretraining set incorporated filtered generated structures from our previous study [42], and controlled experiments confirmed their positive contributions to both pretraining quality and downstream performance (Table S4).

**FIGURE 4:**
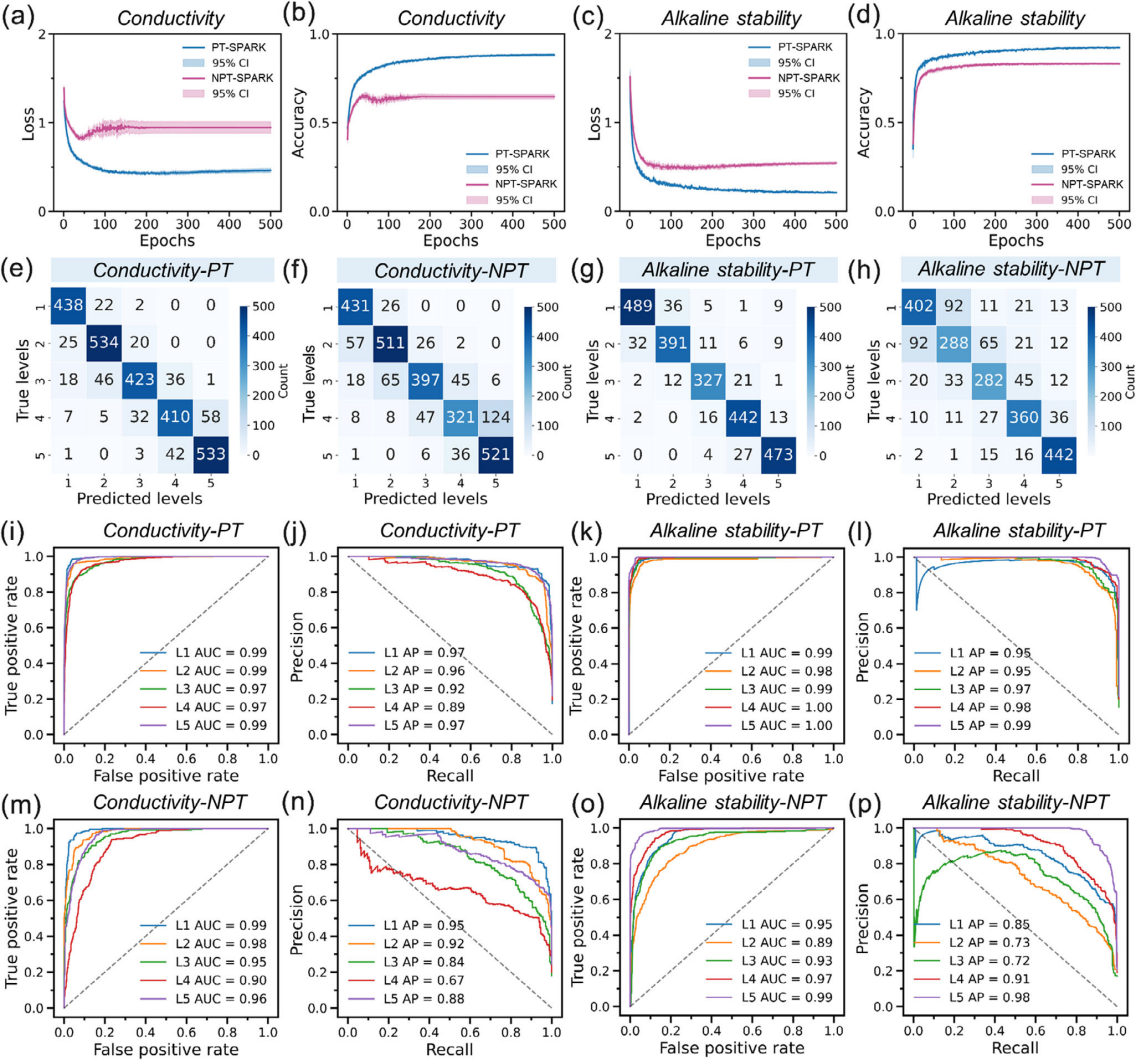
Effect of SPARK pretraining on the grading of hydroxide conductivity and alkaline stability. (a–d) Validation learning curves (loss and accuracy) averaged over 10 independent runs, with shaded 95% confidence intervals; (e–h) Confusion matrices on the test set; (i–p) Receiver operating characteristic curve (ROC) and precision‐recall curve (PR) on the test set, with corresponding area under the ROC curve (AUC) and average precision (AP) values. (NPT and PT denote the non‐pretrained and pretrained models, respectively).

To quantify the gains from pretraining, pretrained SPARK (PT‐SPARK) is compared with its non‐pretrained counterpart (NPT‐SPARK) across grade‐prediction tasks (Figure 4; Tables S5–S6). For conductivity grading (Figure 4a,b), PT‐SPARK converges smoothly, achieving a test loss of 0.4658 (Table S5) and a balanced accuracy (BA) of 0.8797 (Table S6). BA is reported as the primary metric rather than conventional accuracy because it provides a more representative assessment of performance on imbalanced datasets. In contrast, NPT‐SPARK exhibits substantial early‐stage oscillations and converges to a higher loss (0.9397) and lower BA (0.6357). These results demonstrate that pretraining reduces model loss by 50.43% and improves BA by 38.38%. For alkaline stability grading (Figure 4c,d), both models display more stable learning curves; however, PT‐SPARK converges faster and achieves superior final performance, reducing the loss from 0.5223 to 0.2078 (a 60.21% reduction) and improving BA from 0.8345 to 0.9152 (a 9.67% improvement). Statistics from ten independent runs are summarized in Table S5, with additional metrics provided in Table S6.

Confusion‐matrix analysis (Figure 4e–h) further indicates that PT‐SPARK misclassifications are largely confined to adjacent grades (±1) for conductivity and stability, indicating reliable grade discrimination. By comparison, NPT‐SPARK exhibits substantially lower accuracy across all grades. The overall discriminative performance and reliability of the models under different classification thresholds were further assessed using receiver operating characteristic curves (ROC) and precision‐recall curves (PR) (Figure 4i–p). PT‐SPARK yields ROC curves approaching the upper‐left corner and PR curves trending toward the upper‐right, with area under the ROC curve (AUC) and average precision (AP) values consistently exceeding 90%, demonstrating strong discriminative capability across all grades. Performance is particularly robust at critical high‐performance tiers (Levels 1–2) for both conductivity and stability. In contrast, NPT‐SPARK shows pronounced deterioration in PR behavior, most notably for conductivity (Figure 4n), where AP drops to 67% for Level 4, indicating poor recognition of this category. A multi‐metric evaluation (Figure S7) corroborates the overall advantage of PT‐SPARK over NPT‐SPARK for both single‐grade and multi‐grade classification, underscoring the effectiveness of pretraining.

**FIGURE 5:**
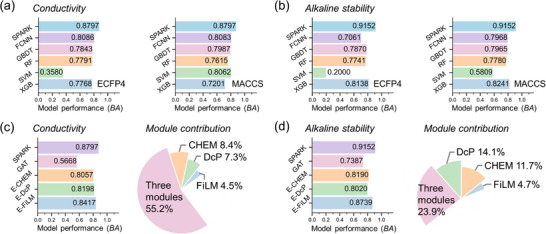
Model performance and ablation analysis: (a, b) comparison of SPARK with conventional machine‐learning models in terms of balanced accuracy (BA); (c, d) Ablation results showing BA for each variant and the relative contribution of each module. (GAT: removal of all three feature modules, retaining only the base graph encoder; E‐CHEM, E‐DcP, and E‐FILM represent the removal of CHEM, the dual‐channel pretraining architecture (DcP), and FiLM module, respectively).

To further validate the superiority of SPARK, its performance is compared against conventional models, including fully connected neural networks (FCNN), random forest (RF), gradient boosting decision trees (GBDT), support vector regression (SVR), and XGBoost (XGB), under two molecular fingerprint representations (MACCS and ECFP4). As shown in Figure 5a,b, Figure S8, and Tables S7–S9, these fingerprint‐model combinations yield broadly comparable performance. The strongest baselines, FCNN‐ECFP4 for conductivity (BA = 0.8086) and XGB–MACCS for stability (BA = 0.8241), remain markedly below SPARK, which achieves BA values of 0.8797 and 0.9152, respectively. Additionally, as FiLM is introduced to encode operating conditions, its out‐of‐range robustness was assessed by a temperature‐boundary test in the conductivity grading task. When samples at ≥80°C were excluded from training and reserved for external evaluation, the model achieved a BA of 0.8863 on the in‐range set (<80°C) and 0.7027 on the unseen high‐temperature set (≥80°C) (Table S10). Despite some degradation, the extrapolation performance remained acceptable, suggesting that FiLM retains meaningful structure–property relationships beyond the training range. For practical applications, the predicted probability distribution is also reported as an indicator of reliability.

### 2.4 Ablation Study

To quantify the contribution of each core component in SPARK, ablation experiments were conducted by selectively removing the CHEM module (E‐CHEM), the dual‐channel pretraining architecture (E‐DcP), the FiLM module (E‐FiLM), and all three modules simultaneously, yielding the base GAT encoder (GAT). For conductivity‐grade prediction (Figure 5c; Tables S11–S13), introducing CHEM improves BA by 8.4% relative to the standard RDKit‐derived molecular graph (RDKit features in Table S14). This gain is attributed to the chemically meaningful prior knowledge encoded by CHEM, which enriches atom and bond representations with task‐relevant semantics. The dual‐channel pretraining architecture of DEGAT further increases BA by 7.3%, indicating that the two‐stream design more effectively disentangles and complements hydrophilic and hydrophobic segment representations. The FiLM module provides an additional 4.5% improvement over direct concatenation of DEGAT embeddings with experimental parameters, underscoring its advantage in condition‐aware feature modulation. Relative to the base GAT encoder, integrating all three modules increases the BA for conductivity grading by 55.3%, underscoring the synergistic gains from this hierarchical design of the three sequential feature‐learning modules.

For stability‐grade prediction, CHEM, DcP, and FiLM improve BA by 11.7%, 14.1%, and 4.7%, respectively (Figure 5d). The pronounced contribution of DcP (14.1%) emphasizes the importance of complementarily capturing hydrophilic and hydrophobic segment features, which together govern alkaline degradation behavior. FiLM consistently contributes more than 4% across tasks. Overall, SPARK outperforms the base GAT encoder by 23.9% BA in stability grading, confirming the necessity of the combined contributions from CHEM, DcP, and FiLM.

### 2.5 Model Interpretation and Chemical Insights

SPARK's strong performance stems from its ability to recognize polymer‐relevant structural features and highlight key functional groups within repeating units. Importantly, SPARK preserves chemical similarity, i.e., repeating units sharing similar backbones and functional motifs tend to receive consistent assessments and contribute in comparable ways to the predicted grades. This chemically consistent behavior supports a more stable structure–performance attribution, reduces reliance on non‐structural factors, and improves robustness to experimental variability. As a result, SPARK provides more reliable guidance across diverse chemistries and test conditions, while offering clearer and more interpretable design cues.

**FIGURE 6:**
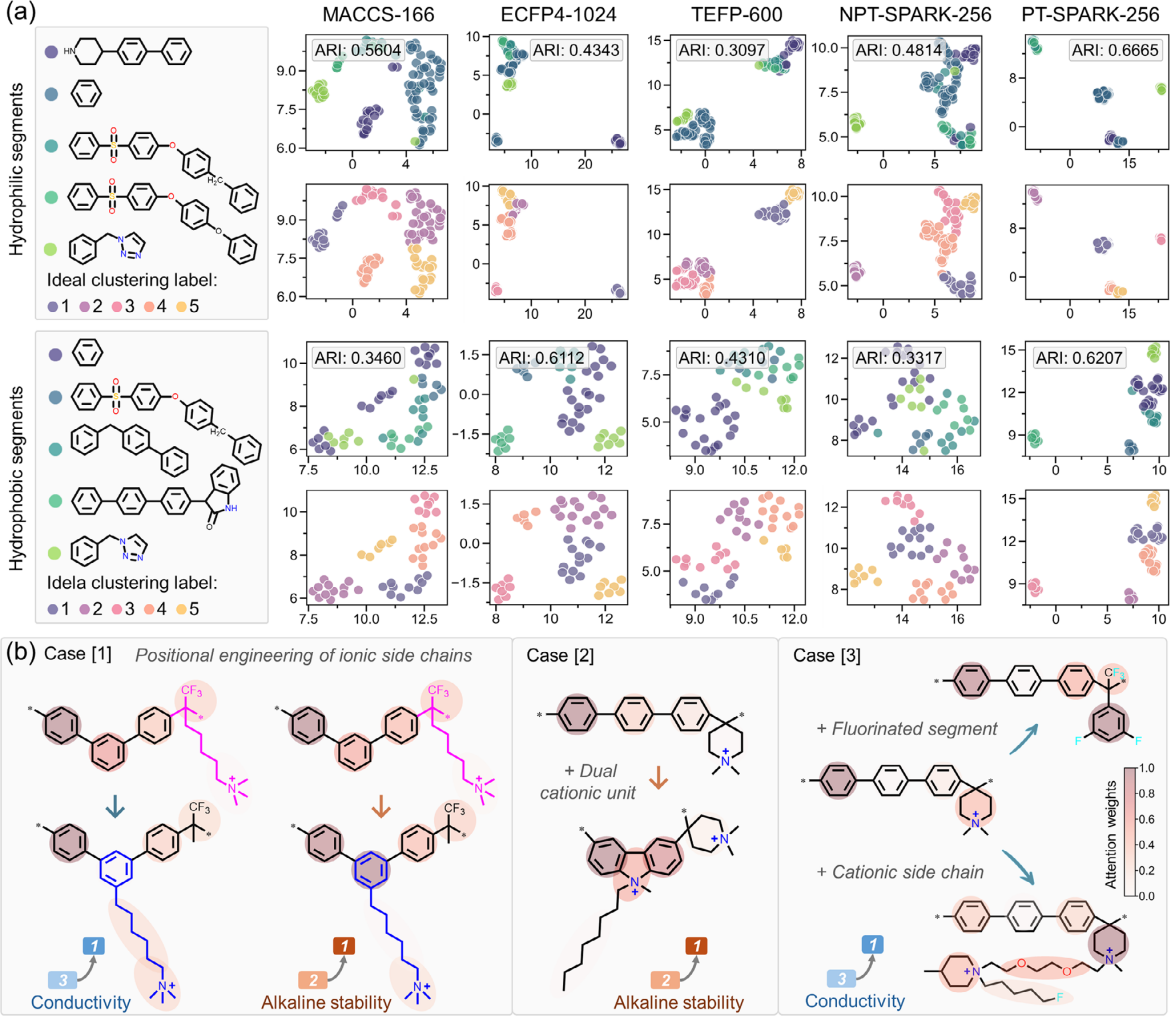
Alignment and interpretability analysis: (a) UMAP visualization of feature representations generated by different methods for AEM repeating units sharing the same scaffold, a higher ARI indicates better clustering separation; ideal clustering labels are obtained by K‐means, headers denote encoder and feature dimension. (b) Region‐level attention maps for three interpretable AEM case studies [43, 44, 45], with normalized final‐layer attention weights; each region contains six atoms to reduce size bias, and property‐grade changes are derived from experimental data.

To evaluate how well different molecular representations preserve structural similarity in chemical space, Uniform Manifold Approximation and Projection (UMAP) was used to visualize the clustering of repeating units sharing the same molecular scaffold. RDKit scaffolds were computed for all AEM repeating units, and structures with identical scaffolds were grouped; the five largest scaffold structures were selected for visualization. As shown in Figure 6a, conventional fingerprints (MACCS, ECFP, and TEFP) and the non‐pretrained model (NPT‐SPARK) exhibit weak scaffold‐wise clustering. That means repeating units sharing the same scaffold are not consistently grouped (poor within‐scaffold cohesion), and different scaffolds show substantial overlap (limited between‐scaffold separation). In contrast, PT‐SPARK maps repeating units with the same scaffold to compact, well‐defined clusters while maintaining clearer separation among different scaffolds. This trend is further supported by the highest adjusted Rand index (ARI) achieved by PT‐SPARK for both hydrophilic and hydrophobic structures, indicating the closest agreement with scaffold‐based partitioning. These results demonstrate that a chemically informed graph combined with dual‐channel pretrained structural representations yields scaffold‐consistent embeddings that better preserve structural similarity in AEM chemical space.

To examine whether the representation advantage of SPARK leads to robust predictive generalization, error distribution, and scaffold‐level transferability were analyzed. No obvious error clustering was found for specific structural motifs, indicating good generalization across diverse chemistries. Larger errors mainly occurred under extreme conditions, especially at 100°C and in alkaline‐stability tests beyond 3000 h, likely owing to limited training data and more complex physicochemical processes. Scaffold‐based validation was also conducted to avoid data leakage from structurally similar polymers. Under this more stringent setting, the BA for conductivity grading decreased only moderately from 0.8734 to 0.8021 (Table S15), confirming reasonably good performance on completely unseen scaffolds. These results indicate that the model learns generalizable AEM structure–property relationships rather than simply memorizing structural templates.

Attention‐based interpretation identifies structural motifs governing ion transport and alkaline degradation, offering actionable guidance for balancing conductivity and stability. Three case studies further demonstrate the interpretability of SPARK by showing how specific structural units drive grade predictions (Figure 6b). In poly(m‐terphenylene alkylene) AEMs, relocating quaternary ammonium groups from the aliphatic segment to the rigid terphenyl backbone increases attention to the cation‐bearing side chain in the conductivity task, suggesting more ordered ion‐conduction sites, while enhanced attention on both cationic units and terphenylene segments in the stability task is consistent with improved shielding against hydroxide attack [43]. In poly(terphenyl‐co‐octylcarbazole‐piperidone), elevated attention to p‐terphenyl and octylcarbazole moieties supports the experimentally observed benefit of the dual‐cation strategy in dispersing charge density and mitigating degradation [44]. In systems containing fluorinated side chains and piperidinium cations, SPARK assigns high attention to both motifs, consistent with their synergistic roles in promoting microphase separation and efficient ion transport [45]. The model outputs after these structural modifications agree well with experimentally observed improvements in conductivity and alkaline‐stability grades (Table S16), with an overall accuracy of 0.875. Overall, these results demonstrate that SPARK not only delivers accurate grade prediction but also provides interpretable, mechanism‐relevant insights that offer transparent and reliable guidance for AEM molecular design and for elucidating complex structure‐property relationships.

### 2.6 Case Study and Validation

**FIGURE 7:**
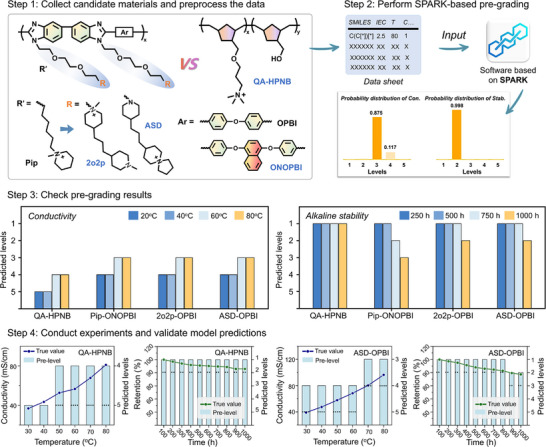
SPARK workflow for pre‐grading and experimental validation of PBI‐ and PNB‐based AEM candidates. Candidate structures were compiled into a datasheet and evaluated by SPARK for property grades. The predictions were used to establish structure–performance trends, prioritize candidates for synthesis, and were then experimentally validated with good agreement to the predicted grades.

A dedicated SPARK‐based software package was developed and validated in a practical AEM design workflow. SPARK was used to pre‐grade a series of to‐be‐synthesized candidates based on polybenzimidazole (PBI) and polynorbornene (PNB) backbones (Figure 7), enabling early‐stage molecular screening. As representative and complementary AEM platforms, PNB provides a highly tunable all‐carbon backbone with high conductivity and good alkaline durability [46, 47], whereas PBI supports multiple ionic groups and ether linkages that favor ionic clustering and more percolated ion‐transport pathways [48, 49]. On this basis, the PBI series was further tailored at both the side‐chain and backbone levels. Side‐chain modifications included flexible two‐ether linkers with hydrophobic terminal tails (2o2p), as well as longer spacers combined with alkaline‐stable N‐spirocyclic functionalities (ASD). Meanwhile, the backbone diphenyl ether unit was replaced by a naphthalene unit to promote segmental stacking (ONOPBI). For comparison, a PNB‐based series enriched with oxygen‐containing segments (ether and hydroxyl groups) was also designed to assess the effects of backbone chemistry and segmental organization on conductivity and stability.

Before SPARK evaluation, candidate materials were curated into a standardized datasheet containing SMILES, repeating‐unit composition (reflecting IEC), and testing conditions. To ensure fair comparison, all candidates were constrained to similar IEC values (≈1.60 mmol/g) under identical conditions. The predicted grades provided an intuitive structure–performance overview. PBI‐based candidates were generally one grade higher in conductivity than PNB‐based candidates, while side‐chain and backbone modifications within the PBI series produced only minor conductivity changes, suggesting that these modifications mainly fine‐tune transport rather than induce large grade transitions. For alkaline stability in 2 M alkaline solution at 80°C, PNB‐based candidates were predicted to remain at Grade 1 over 1000 h, whereas PBI‐based candidates showed more varied degradation behavior depending on structural motifs. Experimental results agreed well with the predicted grades, confirming that SPARK can efficiently prioritize promising candidates for synthesis and testing. Additionally, the model showed strong engineering performance for large‐scale virtual screening. Its lightweight architecture (∼0.5–1.0 million parameters; ∼2–4 MB) supports efficient inference, enabling 10,000 candidate polymers to be screened in ∼20 s on mainstream GPU hardware (excluding I/O overhead under batch inference with asynchronous data loading), which highlights its practical potential.

## 3 Conclusion

A chemically informed dual‐channel graph attention network is established to decode the multiscale structure–property–condition relationships governing hydroxide conductivity and alkaline stability in AEMs. By embedding chemical prior knowledge into molecular graphs, disentangling hydrophilic ionic and hydrophobic non‐ionic segments to capture microphase separation, and incorporating operating conditions via the FiLM mechanism, SPARK enables robust property‐grade prediction. The resulting five‐level classifiers achieve balanced accuracies of 0.8797 for conductivity grading and 0.9152 for stability grading, outperforming conventional machine‐learning baselines. Moreover, attention‐based interpretation highlights functional segments associated with ion‐transport pathways and alkaline degradation, providing mechanistic insight and actionable design guidance. Finally, SPARK is experimentally validated, and the accompanying software package is publicly released to facilitate broader adoption and data‐driven AEM design. Overall, SPARK offers a general and extensible paradigm for polymeric membranes, supporting reliable virtual screening and accelerating the development of next‐generation membrane materials for electrochemical energy conversion.

The authors have cited additional references within the Supporting Information [50, 51]. The SPARK‐based software package associated with this work is available on GitHub at: https://github.com/CH324955/SPARK‐AEM.

## Conflicts of Interest

The authors declare no conflicts of interest.

## Supporting information




**Supporting File**: advs74971‐sup‐0001‐SuppMat.pdf.

## Data Availability

The data that support the findings of this study are available from the corresponding author upon reasonable request.
