# Supplementary material for: Decoding Structure‐Property Relationships in Anion Exchange Membranes via a Chemically Informed Dual‐Channel Graph Attention Network
Source: Adv Sci (Weinh). 2026 Apr 2;13(32):e74971. doi: 10.1002/advs.74971 (PMC13252622; doi:10.1002/advs.74971)
Supplement: Supplementary file 1 — Supporting File: advs74971‐sup‐0001‐SuppMat.pdf. [file ADVS-13-e74971-s001.pdf]

Supporting Information

Decoding Structure-Property Relationships in Anion Exchange Membranes via a Chemically Informed Dual-Channel Graph Attention Network

Wanting Chen,[a] *Ye Hu,*[a] *Zijun Xiao,*[b] *Deming Xia,*[b] *Bo Pang,*[a] *Xuemei Wu,*[a] *Gaohong He* [a,] *

[a] State Key Laboratory of Fine Chemicals, Frontier Science Center for Smart Materials, School of Chemical Engineering, Dalian University of Technology, Dalian, 116024, China

[b] Key Laboratory of Industrial Ecology and Environmental Engineering (MOE), Dalian Key Laboratory on Chemicals Risk Control and Pollution Prevention Technology, School of Environmental Science and Technology, Dalian University of Technology, Dalian 116024, China

*Gaohong He: hgaohong@dlut.edu.cn

1. Methods

1.1 CHEM Module

The CHEM module converts the input SMILES strings into molecular graphs with node/edge embeddings, while progressively incorporating chemical prior knowledge to yield physically interpretable, task-adaptive molecular representations. Diverse priors are encoded using four complementary schemes (Table S1).

Pretrained embedding networks are adopted for high-cardinality discrete features (e.g., atom type, bond type, hybridization state). Specifically, atomic numbers are mapped to 32-dimensional vectors using the GROVER atom-embedding table,[50] which encodes continuous periodic trends (such as the electronegativity variations from alkali metals to halogens) and correlated chemical behaviors across elements. Bond types are embedded using NGG,[51] whose contrastive pretraining learns nonlinear structure–reactivity relationships, distinguishing, for instance, single bonds from conjugated double bonds in terms of electron delocalization.

One-hot encoding is reserved for low-cardinality, mutually exclusive categorical features (e.g., formal charge, chirality labels, stereochemical configuration). A 3-dimensional one-hot vector for chirality (R/S/achiral) directly encodes absolute differences in molecular spatial configuration. Formal charges (−1/0/+1) are also one-hot encoded to rigidly separate charged centers from neutral atoms, which is particularly important for accurately identifying ion-transport sites in AEMs.

Raw-value embedding is applied to continuous physico-chemical parameters such as electronegativity, ionization energy, and van der Waals radius. After numerical normalization, these

scalar features are directly fed into the network to preserve their intrinsic ordering and nonlinear response characteristics.

Bucket embedding acts as a hybrid strategy for features that combine an underlying continuous distribution with characteristic thresholds (e.g., the number of hydrogen bonds). For instance, the hydrogen-bond donor count is discretized into four bins [0, 1, 2, ⩾3], corresponding to “no donor”, “single donor”, “double donor”, and “multi-donor competition” states, which are then mapped to an 8-dimensional embedding. Such binning mitigates the over-smoothing of sharp threshold effects by pure numerical embeddings (e.g., conformational reorganization that may arise upon forming a third hydrogen bond), while avoiding the loss of continuous trends that would result from a purely one-hot treatment.

1.2 DEGAT framework

A standardized workflow for polymer structure parsing was established. Homopolymer repeat units were directly extracted from SMILES with [*] marking the connection sites, while comonomer units, side chains, crosslinking motifs, and compositional ratios were explicitly encoded for more complex polymers. To clarify this process, Fig. S9 presents a representative example showing the mapping from the original literature description to the final model input. To separately learn the representations of hydrophilic and hydrophobic AEM segments, a dual-channel edge-enhanced graph attention (DEGAT) framework was adopt. DEGAT is first pretrained in a self-supervised manner to obtain optimal initialization of the encoder weights (Table S2). Specifically, we apply adoptededededdom masking strategy in which 30% of node embedding vectors in the input graph are set to zero. The masked graph is then passed through stacked GAT layers to yield node-level graph

embeddings, which are subsequently fed into a three-layer multilayer perceptron (MLP) decoder to reconstruct the original features of the masked nodes. Pretraining is accomplished by minimizing the discrepancy between the reconstructed and original features of all masked nodes. The detailed architecture of DEGAT is illustrated in Fig. S1, and the message-passing equations of the GAT Layer used in SPARK are given in Section 1.3.

Two channels, DEGAT-A and DEGAT-B, are proposed with identical architectures and initial parameters, but are pretrained on different structural subsets. Structural units are first partitioned into hydrophilic and hydrophobic segments based on their total formal charge: units with zero total charge are treated as hydrophobic and used to pretrain DEGAT-B, whereas all remaining (charged) units are used to pretrain DEGAT-A (Fig. S2). During pretraining, when the model performance shows no appreciable improvement for 20 consecutive epochs, the corresponding parameters are saved as the optimal weights. Because the task involves predicting continuous node features, the loss function is expressed as the mean squared error (MSE) as follows:

$$\mathcal{L}_{\mathrm{rec}} = \frac{1}{|M|}\sum_{i\in M}\sum_{d=1}^{d_{\mathrm{atom}}}(\hat{x}_{i,d} - x_{i,d})^2$$

where $\mathcal{L}_{\mathrm{rec}}$ represents the reconstruction loss; M denotes the set of masked nodes ($M = \{i \mid \text{Node } i \text{ is masked}\}$); $\hat{x}_{i,d} \in \mathbb{R}^{d_{\mathrm{atom}}}$ and $x_{i,d} \in \mathbb{R}^{d_{\mathrm{atom}}}$ are the the predicted and original feature vectors of the i-th masked node, respectively; d_{atom} is the atomic embedding dimension and set to 103.

The self-supervised pretraining dataset for DEGAT comprises AEM repeating-unit structures collected from the literature, supplemented with additional candidates generated by our previously developed generative model.[42] Generated structures are screened for both chemical validity and

synthesizability before being integrated with the experimental set. Molecular plausibility is evaluated by computing the Tanimoto similarity of each generated structure against all experimentally reported compounds; a generated molecule is retained only if its maximum Tanimoto similarity to at least one real structure exceeds a predefined threshold (e.g., >5). Synthesizability is quantified using the synthetic accessibility (SA) score, and only candidates with SA ≤5 are kept. Representative SA scores and Tanimoto coefficients for selected structures are provided in Table S3 as illustrative examples. In the final pretraining dataset, ionic (hydrophilic) and non-ionic (hydrophobic) units are assigned to the two DEGAT channels separately. The ionic subset comprises 19,063 units (540 experimental and 18,523 generated), whereas the non-ionic subset comprises 3,293 units (141 experimental and 3,152 generated).

1.3 Computational procedure of SPARK

1.3.1 DEGAT

The molecular input to DEGAT consists of node and edge features. The node feature is $H \in \mathbb{R}^{N \times d_{\mathrm{knot}}}$, where d_{knot} is the atomic embedding dimension and is equal to 103; the edge feature is $E \in \mathbb{R}^{M \times d_{edge}}$, where d_{edge} is the bond embedding dimension and is set to 18. As edges are treated as bidirectional and self-loops are included, the edge index matrix is $I \in \mathbb{R}^{2 \times (2M+N)}$.

Consider two connected nodes a and b with node embeddings $\vec{h}_a$ and $\vec{h}_b$, respectively. The raw attention coefficient from node a to node b is defined as:

$$\alpha_{ab} = \mathrm{LeakyReLU}(\mathbf{a}^{\mathrm{T}}[\mathbf{W}_a \vec{h}_a \,||\, \mathbf{W}_b \vec{h}_b \,||\, \mathbf{U} E_{ab}])$$

where LeakyReLU is the activation function, ‖ denotes vector concatenation, E_{ab} is the bond embedding between nodes a and b, $\mathbf{a}$, $\mathbf{W}_{\mathrm{a}}$, $\mathbf{W}_{\mathrm{b}}$, and $\mathbf{U}$ are learnable weight matrices/vectors that are

optimized during backpropagation. For node a, the normalized attention coefficients over all its neighbors (including itself) are obtained via a softmax operation. Extending this to the entire molecular graph and the k-th attention head in a multi-head setting yields:

$$\alpha_{ij}^{(k)} = \mathrm{softmax}(a_{ij}^{(k)})$$

where i and j index all connected node pairs in the graph and denotes the k-th attention head. The node feature aggregation for the k-th head is then given by:

$$h_i^{(k)} = \sum_{j \in N(i)} \alpha_{ij}^{(k)} \tilde{h}_j^{(k)}$$

N(i) denotes the neighborhood of node i(including i itself); $\tilde{h}_j^{(k)}$ is the projected embedding of node j. Collecting all nodes yields the attention-based representation $h_j^{(k)}$ for head k. Multi-head attention is realized by concatenating the outputs of all heads. With four attention heads used in this work, the concatenated representation is:

$$\mathbf{H}' = \Vert_{k=1}^{4} \mathbf{H}^{(k)} \in \mathbb{R}^{N \times d_{\mathrm{out}}}$$

$$\mathbf{H}^{(k)} \in \mathbb{R}^{N \times (d_{\mathrm{out}}/4)}$$

where d_{out} is the output dimension, $\mathbf{H}'$ is the output of GATConv. The GATConv output is then passed through a residual projection:

$$\mathbf{H}_{\mathrm{res}} = \mathbf{H}^{(l\text{-}1)}\mathbf{W}_{\mathrm{res}} + \mathbf{b}_{\mathrm{res}}$$

$$\mathbf{W}_{\mathrm{res}} \in \mathbb{R}^{d_{\mathrm{in}} \times d_{\mathrm{out}}}$$

where $\mathbf{W}_{\mathrm{res}}$ and $\mathbf{b}_{\mathrm{res}}$ are the learnable weight matrix and bias vector of the residual connection, respectively; and l indexes the current layer.

The final output of the l-th GAT layer is obtained by applying activation, dropout, and layer normalization of $\mathbf{H}_{\mathrm{res}}$:

$$\mathbf{H}^{(l)} = \text{LayerNorm}(\text{ELU}(\text{Dropout}(\mathbf{H}')) + \mathbf{H}_{\text{res}})$$

where ELU is the activation function, Dropout denotes random weight dropout, LayerNorm is the layer normalization operator. Stacking multiple GAT layers follows the same procedure.

For the classification tasks, the loss function is the cross-entropy loss, defined as:

$$\mathcal{L} = -\frac{1}{\text{N}}\sum_{i=1}^{N}\log(\frac{\exp(x_{i,\text{true}})}{\sum_{c=1}^{C} exp(x_{i,c})})$$

where $x_{i,c}$ denotes the logit corresponding to class c, C is the total number of classes and is equal to 5, and N is the batch size.

1.3.2 Grade-prediction models

In the downstream classification task, the pretrained DEGAT-A and DEGAT-B encoders are further optimized via full fine-tuning, using their pretrained weights as initialization to preserve and refine the dual-channel representations. Hydrophilic and hydrophobic structural repeating units are encoded by DEGAT-A and DEGAT-B, respectively, to obtain latent embeddings that are concatenated and passed through a FiLM-based fusion module, enabling coordinated alignment and weighted modulation of multi-scale features. The fused representation is then fed into a three-layer residual dense classification head, which outputs the class-probability distribution for each sample and is trained using a cross-entropy loss.

For AEM molecules comprising more than two structural repeating units, a primary hydrophilic segment and a primary hydrophobic segment are first selected based on their composition ratio (the unit with the larger ratio is designated as primary; when two segments exhibit identical ratios, one unit is randomly chosen). These primary segments are then encoded by the dual-channel DEGAT-A/B encoders. The remaining segments are routed to DEGAT-A and DEGAT-B according to their

hydrophilic or hydrophobic character to obtain auxiliary embeddings, which are subsequently fused with the corresponding primary embeddings to enhance the hydrophilic or hydrophobic signatures of the overall molecular representation. The resulting enriched embeddings are finally passed to the FiLM and multi-scale fusion module (Fig. S2).

For the ionic-conductivity grading task, only the test temperature (T) is incorporated into the representation via the FiLM mechanism as follows:

$$[\boldsymbol{\gamma}_c;\boldsymbol{\beta}_c] = \mathrm{MLP}(\mathrm{Norm}(T)) \in \mathbb{R}^d$$

$$\mathbf{f}_c = [\vec{a};\vec{b}] \in \mathbb{R}^d$$

$$\mathbf{f}_{\mathrm{cmod}} = \gamma_c \odot f_c + \beta_c \in \mathbb{R}^d$$

where the subscript c denotes the conductivity grading task; $\boldsymbol{\gamma} \in \mathbb{R}^d$ and $\boldsymbol{\beta} \in \mathbb{R}^d$ are the feature scaling factor and bias term, respectively, obtained by mapping the normalized temperature T through two multilayer perceptrons (MLPs) with ReLU activations; d corresponds to the sum of the output dimensions of DEGAT-A and DEGAT-B; **f** is the concatenated graph-attention embedding of molecules A and B; the fused feature $\mathbf{f}_{\mathrm{cmod}}$ is thus obtained by applying the temperature-dependent scaling and bias to **f**; $\odot$ denotes element-wise multiplication.

In the alkaline-stability grading task, the test duration (T_{r}), test temperature (T_{As}) and alkaline concentration (C) are incorporated via the FiLM mechanism, expressed as:

$$\mathbf{c} = [\mathrm{Norm}(T_{\mathrm{r}}); \mathrm{Norm}(T_{\mathrm{As}}); \mathrm{Norm}(C)]$$

$$[\boldsymbol{\gamma}_{\mathrm{As}};\boldsymbol{\beta}_{\mathrm{As}}] = \mathrm{MLP}(\mathbf{c}) \in \mathbb{R}^{2d}$$

$$\mathbf{f}_{\mathrm{As}} = [\vec{a};\vec{b}] \in \mathbb{R}^d$$

$$\mathbf{f}_{\mathrm{Asmod}} = \boldsymbol{\gamma}_{\mathrm{As}} \odot \mathbf{f}_{\mathrm{As}} + \boldsymbol{\beta}_{\mathrm{As}} \in \mathbb{R}^d$$

where the subscripts r and As both refer to the alkaline-stability grading task. The context vector **c** is formed by normalizing T_{As} and C, and then concatenating them; **c** is subsequently mapped through an MLP to yield the modulation parameters $\boldsymbol{\gamma}$ and $\boldsymbol{\beta}$; the vector f denotes the concatenated graph-attention embedding of molecules A and B; the fused feature is denoted as $\mathbf{f}_{\mathrm{Asmod}}$, which is thus obtained by applying the weights and biases derived from T_{r}, T_{As} and C to the original feature **f**.

1.4 Experimental setup

For the self-supervised pretraining dataset, the database was randomly split into training and validation subsets with a ratio of 8:2. For downstream fine-tuning, the labeled dataset was randomly partitioned into training/validation/test subsets in a ratio of 7:1.5:1.5. Each fine-tuning task was repeated 10 times with different random seeds controlling the data splits. All chemical structures of repeating units were represented as SMILES strings and processed using the open-source cheminformatics toolkit RDKit to construct molecular graphs, including node features and edge indices. Detailed hyperparameter settings for both pretraining and fine-tuning are summarized in Table S2. SPARK was implemented in PyTorch and trained on an Ubuntu workstation equipped with an NVIDIA GeForce RTX 3080 Ti GPU.

1.5. Calculation of Model Performance

(1) Feature-reconstruction performance

The feature-reconstruction performance of DEGAT during pretraining is evaluated using the mean squared error (MSE) and the coefficient of determination (R^2) as follows:

$$\mathrm{MSE} = \frac{1}{\mathrm{n}}\sum_{i=1}^{n}(y_i - \hat{y}_i)^2$$

$$R^2 = 1 - \frac{\sum_{i=1}^{n}(y_i - \hat{y}_i)^2}{\sum_{i=1}^{n}(y_i - \hat{y}_i)^2}$$

where y_i and $\hat{y}_i$ denote the actual and predicted values for the i sample, respectively; and n represents the total number of samples.

(2) Per-grade classification performance

Per-grade performance is evaluated using the following metrics: Precision_i, balanced accuracy (BA_i), Recall_i, F1-Score ($F_{1,i}$), Specificity_i, Negative predictive value (NPV_i), False positive rate (FPR_i), False negative rate (FNR_i), Receiver operating characteristic curve (ROC_i), Area under the ROC curve (AUC_i). Here, the subscript i (i=1, 2, 3, 4, 5) denotes the specific class level. Their definitions were summarized in Table S17.

Precision_i measures the exactness of the predictions by calculating the proportion of actual positive instances among all instances predicted as positive:

$$\text{Precision}_i = \frac{\text{TP}_i}{\text{TP}_i + \text{FP}_i}$$

where True Positive (TP_i) denotes the number of instances correctly predicted as positive, and False Positive (FP_i) represents the number of instances incorrectly predicted as positive.

Recall indicates the proportion of actual positive instances that are correctly identified, reflecting the model's capability to capture positive cases:

$$\text{Recall}_i = \frac{\text{TP}_i}{\text{TP}_i + \text{FN}_i}$$

where False Negative (FN_i) denotes the number of instances incorrectly predicted as negative. It is important to note that in this study, the negative class is defined as the aggregation of the remaining four non-target classes.

$F_{1,i}$ represents the harmonic mean of Precision_i and Recall_i, serving to balance the trade-off between these two metrics:

$$F_{1,i} = \frac{2 \times \text{Precision}_i \times \text{Recall}_i}{\text{Precision}_i + \text{Recall}_i}$$

Specificity_i measures the proportion of actual negative instances that are correctly identified as negative, reflecting the model's capability to reject negative samples:

$$\text{Specificity}_i = \frac{\text{TN}_i}{\text{TN}_i + \text{FP}_i}$$

where TN_i (True Negative) denotes the number of instances correctly predicted as negative.

NPV_i represents the proportion of actual negative instances among all instances predicted as negative, serving to enhance the confidence in negative predictions:

$$\text{NPV}_i = \frac{\text{TN}_i}{\text{TN}_i + \text{FN}_i}$$

BA_i is defined as the arithmetic mean of Recall_i and Specificity_i. This metric is employed to mitigate the bias arising from disparities in sample sizes across different classes and is calculated by:

$$\text{BA}_i = \frac{\text{Recall}_i + \text{Specificity}_i}{2}$$

FPR_i (False positive rate) represents the proportion of actual negative instances incorrectly predicted as positive, reflecting the risk of false alarms. Conversely, FNR_i (False negative rate) denotes the proportion of actual positive instances incorrectly predicted as negative, indicating the risk of missed detections. Their formulas are expressed as:

$$\text{FPR}_i = \frac{\text{FP}_i}{\text{TN}_i + \text{FP}_i}$$

$$\text{FNR}_i = \frac{\text{FN}_i}{\text{FN}_i + \text{TP}_i}$$

Furthermore, ROC_i refers to the curve plotted by mapping the True Positive Rate (TPR) against the False Positive Rate (FPR) at varying decision thresholds, while AUC_i represents the area under the ROC_i curve.

(3) Overall multi-class classification performance

For the grading tasks, overall performance is mainly assessed using Accuracy (Acc), balanced accuracy (BA), macro-averaged F1-score ($F_{1,\ \mathrm{macro}}$), and macro-averaged area under the precision–recall curve (AP_{macro}). Acc represents the proportion of correctly classified samples among all samples, reflecting the global classification accuracy. BA is computed as the average over classes to mitigate the impact of class imbalance. $F_{1,\mathrm{macro}}$ provides a harmonic measure balancing precision and recall across all classes, while AP_{macro} evaluates the robustness of the model in identifying minority classes. The following metrics serve as supplements for evaluating the overall performance of the model: Macro-average Precision (P_{macro}), Macro-average Recall (R_{macro}), Micro-average F1-score ($F_{1,\mathrm{micro}}$), Micro-average Precision (P_{micro}), Micro-average Recall (R_{micro}), Cohen's Kappa coefficient (κ), Macro AUC-ROC (AR_{macro}). Their definitions were summarized in Table S18. All grading tasks are repeated ten times with different random seeds, and corresponding confidence intervals (CIs) are reported.

Specifically, Acc represents the ratio of the total number of correctly predicted instances to the total number of instances. The formula is expressed as:

$$\mathrm{Accuracy} = \frac{\mathrm{TP} + \mathrm{TN}}{\mathrm{TP} + \mathrm{TN} + \mathrm{FP} + \mathrm{FN}}$$

While TP, TN, FP, FN, and FN share the same fundamental meanings as their class-specific counterparts (TP_i, TN_i, FP_i and FN_i), their calculation scopes differ. Specifically, the latter are

computed individually for each class, whereas the former are derived from the aggregated prediction results. Furthermore, BA is employed here to assess the overall performance. It is defined as the arithmetic mean of recall scores across all classes. The formula is expressed as:

$$\mathrm{BA} = \frac{1}{5}\sum_{i=1}^{5}\mathrm{recall}_i$$

P_{macro}, R_{macro} and $F_{1,\mathrm{macro}}$ denote the macro-averaged Precision, Recall, and F_1, respectively, while P_{micro}, R_{micro}, and $F_{1,\mathrm{micro}}$ represent their micro-averaged counterparts. Specifically, the macro-average is calculated as the arithmetic mean, treating all classes equally regardless of sample size. In contrast, the micro-average is derived by globally aggregating the TP, TN, FP, and FN counts across all categories, rendering it more sensitive to the performance on majority classes. Combining both macro- and micro-averaged metrics facilitates a comprehensive assessment of the overall predictive performance. Their formulas are expressed as:

$$P_{\mathrm{macro}} = \frac{1}{5}\sum_{i=1}^{5}\frac{\mathrm{TP}_i}{\mathrm{TP}_i + \mathrm{FP}_i}$$

$$R_{\mathrm{macro}} = \frac{1}{5}\sum_{i=1}^{5}\frac{\mathrm{TP}_i}{\mathrm{TP}_i + \mathrm{FN}_i}$$

$$F_{1,\,\mathrm{macro}} = \frac{1}{5}\sum_{i=1}^{5}\frac{2 \times \mathrm{Precision}_i \times \mathrm{Recall}_i}{\mathrm{Precision}_i + \mathrm{Recall}_i}$$

$$P_{\mathrm{micro}} = \frac{\sum_{i=1}^{5}\mathrm{TP}_i}{\sum_{i=1}^{5}(\mathrm{TP}_i + \mathrm{FP}_i)}$$

$$R_{\mathrm{micro}} = \frac{\sum_{i=1}^{5}\mathrm{TP}_i}{\sum_{i=1}^{5}(\mathrm{TP}_i + \mathrm{FN}_i)}$$

$$F_{1,\,\mathrm{micro}} = 2 \times \frac{P_{\mathrm{micro}} \times R_{\mathrm{micro}}}{P_{\mathrm{micro}} + R_{\mathrm{micro}}}$$

κ (Cohen's Kappa) is employed to evaluate the agreement between the classification results and the ground truth, explicitly correcting for the agreement that could occur by mere chance (random guessing). The formula is expressed as:

$$\kappa = \frac{P_o - P_e}{1 - P_e}$$

where $P_o = \frac{\sum_{i=1}^{5} \mathrm{TP}_i}{\mathrm{N}}$ represents the observed agreement and N denotes the total number of samples. P_e stands for the expected agreement by chance. The formula is expressed as:

$$P_e = \sum_{i=1}^{5} \left(\frac{\mathrm{Real\_distribution}_i}{\mathrm{N}} \times \frac{\mathrm{Pre\_distribution}_i}{\mathrm{N}}\right)$$

Finally, $\mathrm{AR_{macro}}$ is defined as the arithmetic mean of the AUC scores calculated for each class. The formula is expressed as:

$$\mathrm{AR_{macro}} = \frac{1}{5} \sum_{i=1}^{5} \mathrm{AUC}_i$$

$\mathrm{AP_{macro}}$ represents the Area Under the Precision-Recall (PR) Curve. This metric is utilized to evaluate the model's comprehensive performance specifically regarding positive instances. The formula is expressed as:

$$\mathrm{AP_{macro}} = \frac{1}{5} \sum_{i=1}^{5} \mathrm{AP}_i$$

AP (Average Precision) corresponds to the area under the Precision-Recall (PR) curve. The PR curve is generated by plotting Precision against Recall at varying decision thresholds.

(4) The impact of the generated data on model performance

To quantify the impact of the generated data on model performance, controlled comparative experiments were conducted. Group A used only literature-derived data for pretraining, whereas Group B used both literature data and the filtered generated data. Both groups adopted the same

model architecture and training hyperparameters, and were compared in terms of pretraining fitting accuracy and conductivity grading accuracy on the same test set.

(5) The adaptability of the FiLM module

To evaluate the adaptability of the FiLM module to operating conditions beyond the training range, a temperature-boundary test was conducted. In the conductivity grading task, all samples measured at temperatures ⩾ 80 °C were excluded from training, and the model was trained and evaluated using only data collected at operating temperatures below 80 °C. The excluded high-temperature samples were then used as an external test set to assess the model's extrapolation performance.

(6) Scaffold-based validation

As model performance may be overestimated when structurally similar polymers appear in both the training and test sets, scaffold-based validation was performed. Specifically, Bemis-Murcko scaffolds were calculated using RDKit, and polymers within the same scaffold family were assigned exclusively to either the training or test set to prevent structural data leakage. Using this grouped split, the model was retrained for the conductivity grading task. In total, 24 scaffold families comprising 1067 data points were held out as the test set to evaluate the model's performance on entirely unseen scaffolds.

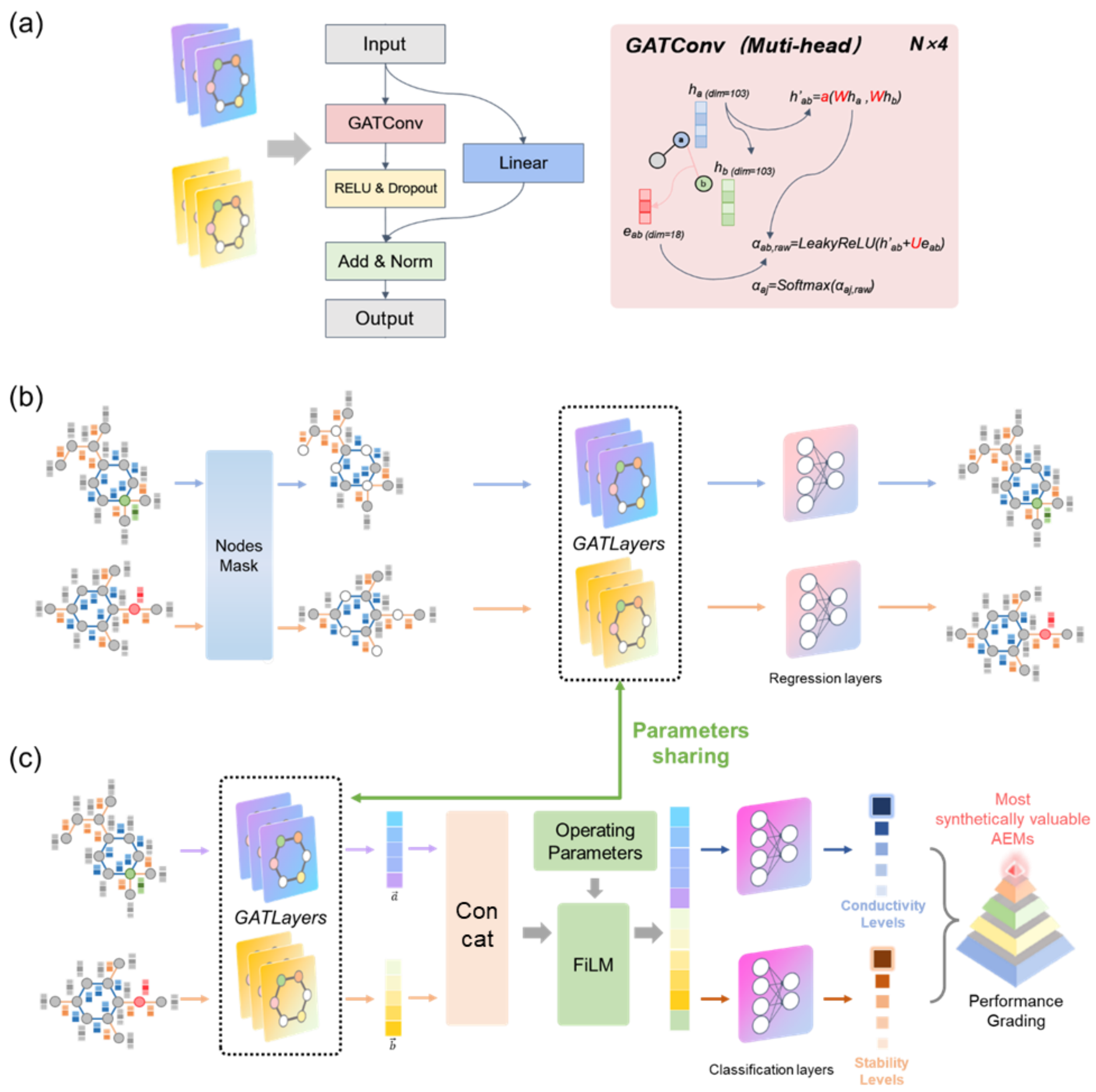

Fig. S1 (a) Detailed architecture and computational flow of the GAT layer in SPARK; (b) self-supervised pretraining of DEGAT via a feature-reconstruction task; (c) fine-tuning of the whole grade-prediction model.

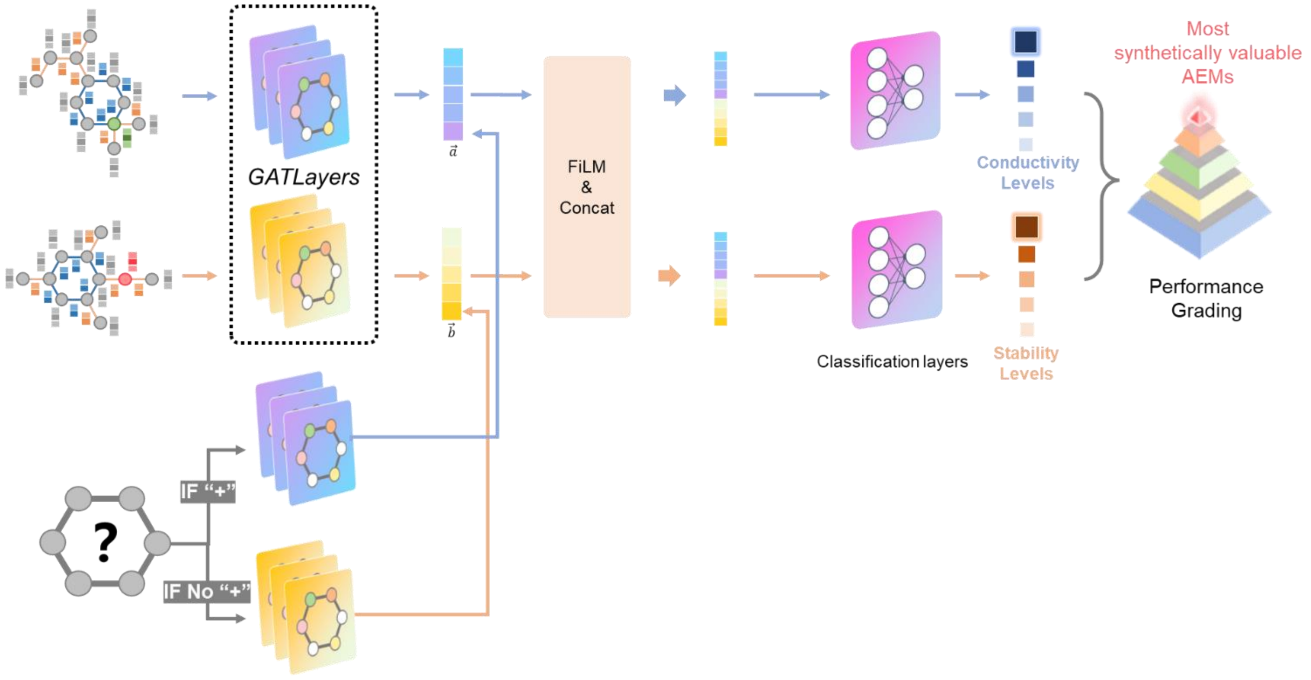

Fig. S2 Computational workflow of SPARK for AEMs comprising more than two repeating units.

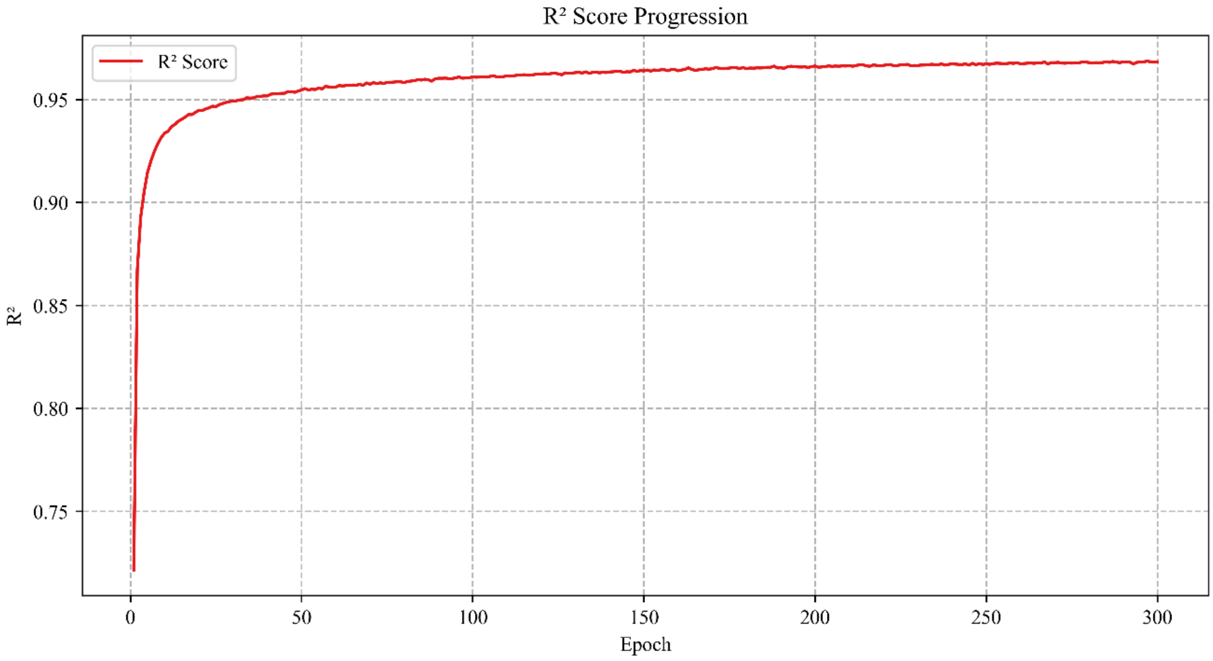

Fig. S3 Pretraining R^2 curve of DEGAT-A for ionic repeating units (SMILES A).

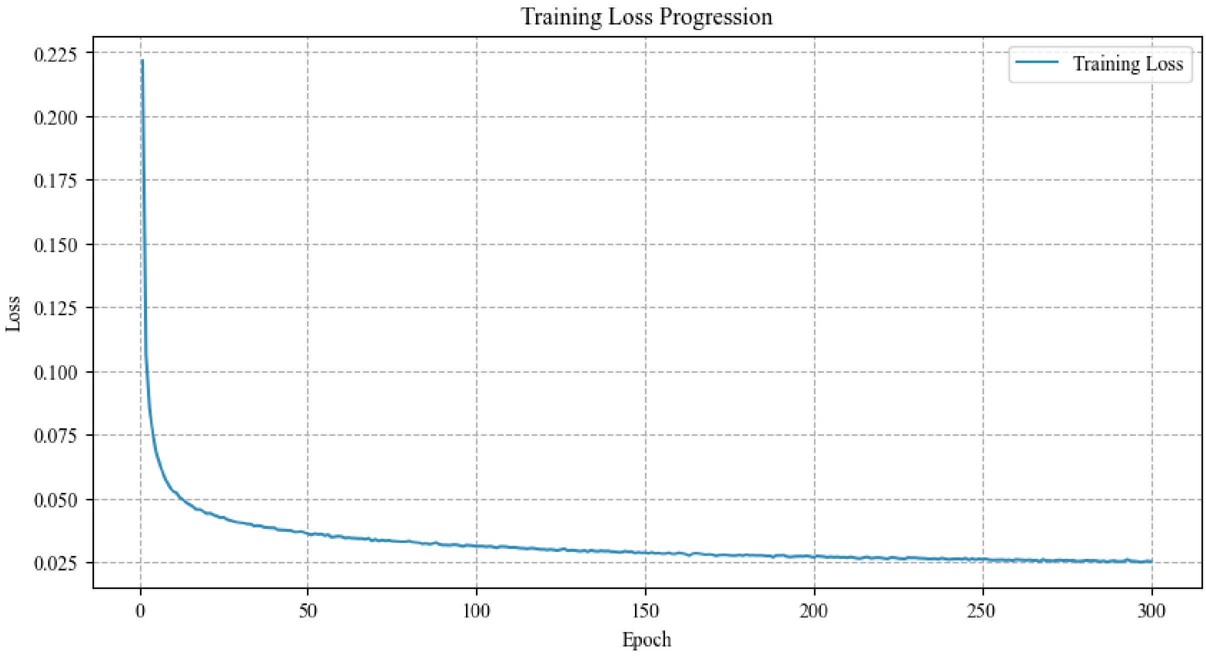

Fig. S4 Pretraining loss curve of DEGAT-A for ionic repeating units (SMILES A).

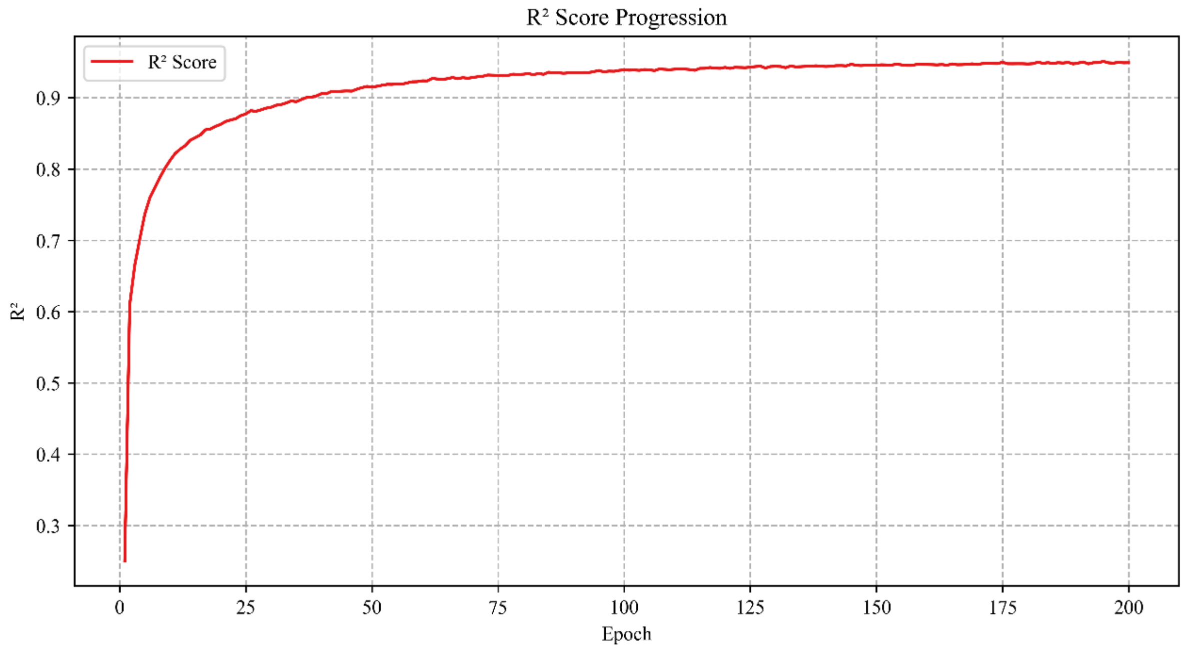

Fig. S5 Pretraining R^2 curve of DEGAT-B for non-ionic repeating units (SMILES B).

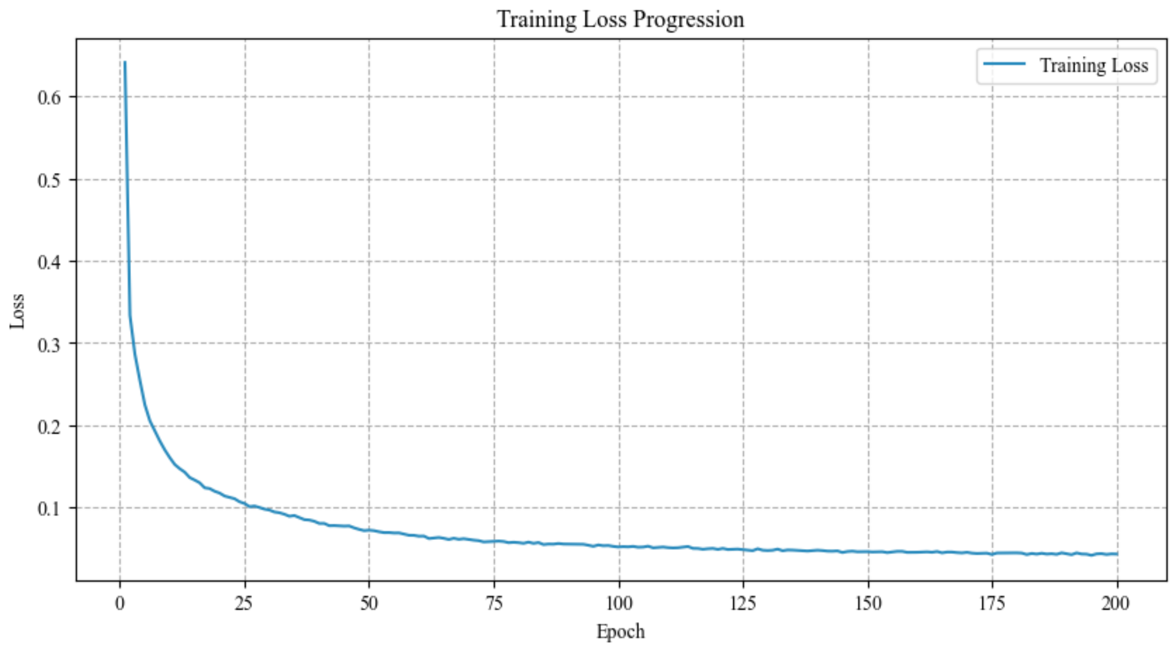

Fig. S6 Pretraining loss curve of DEGAT-B for non-ionic repeating units (SMILES B).

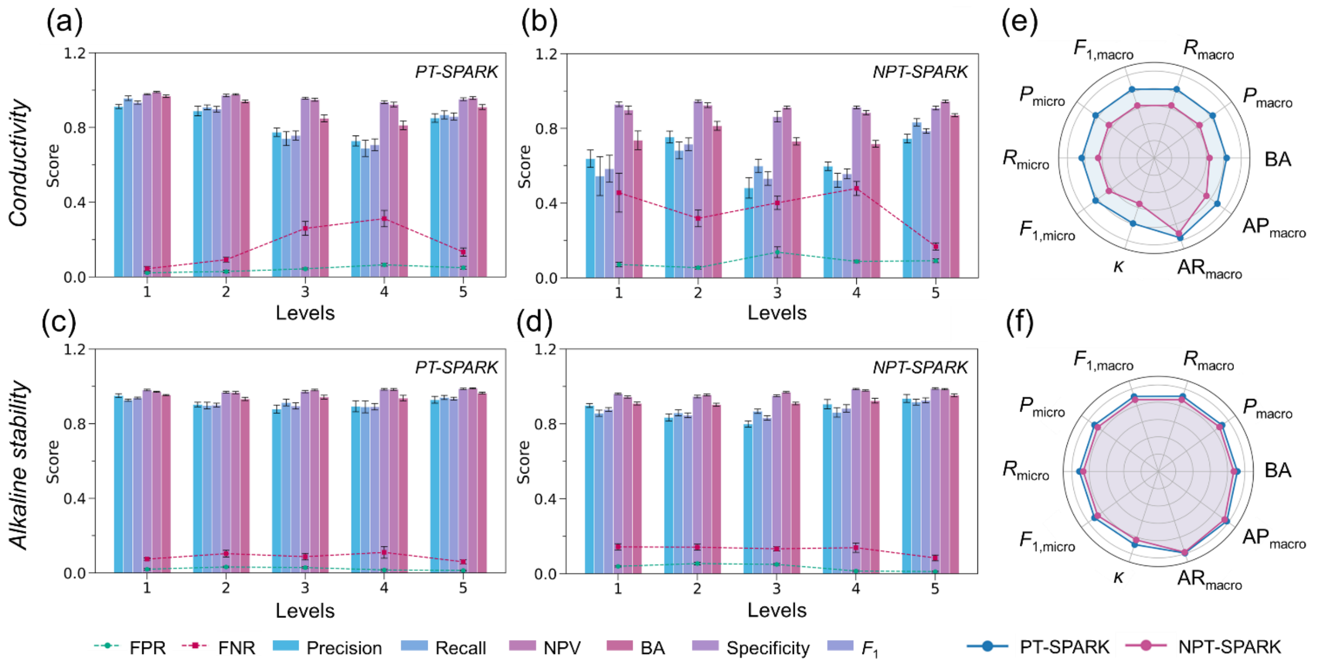

Fig. S7 Supplementary comparison of pretrained (PT-SPARK) and non-pretrained (NPT-SPARK) models: (a, b) OH^--conductivity grading performance; (c,d) alkaline-stability grading performance; (e,f) radar-chart comparison across multiple metrics. Panels (a, b, e) correspond to conductivity, and panels (c, d, f) correspond to alkaline stability.

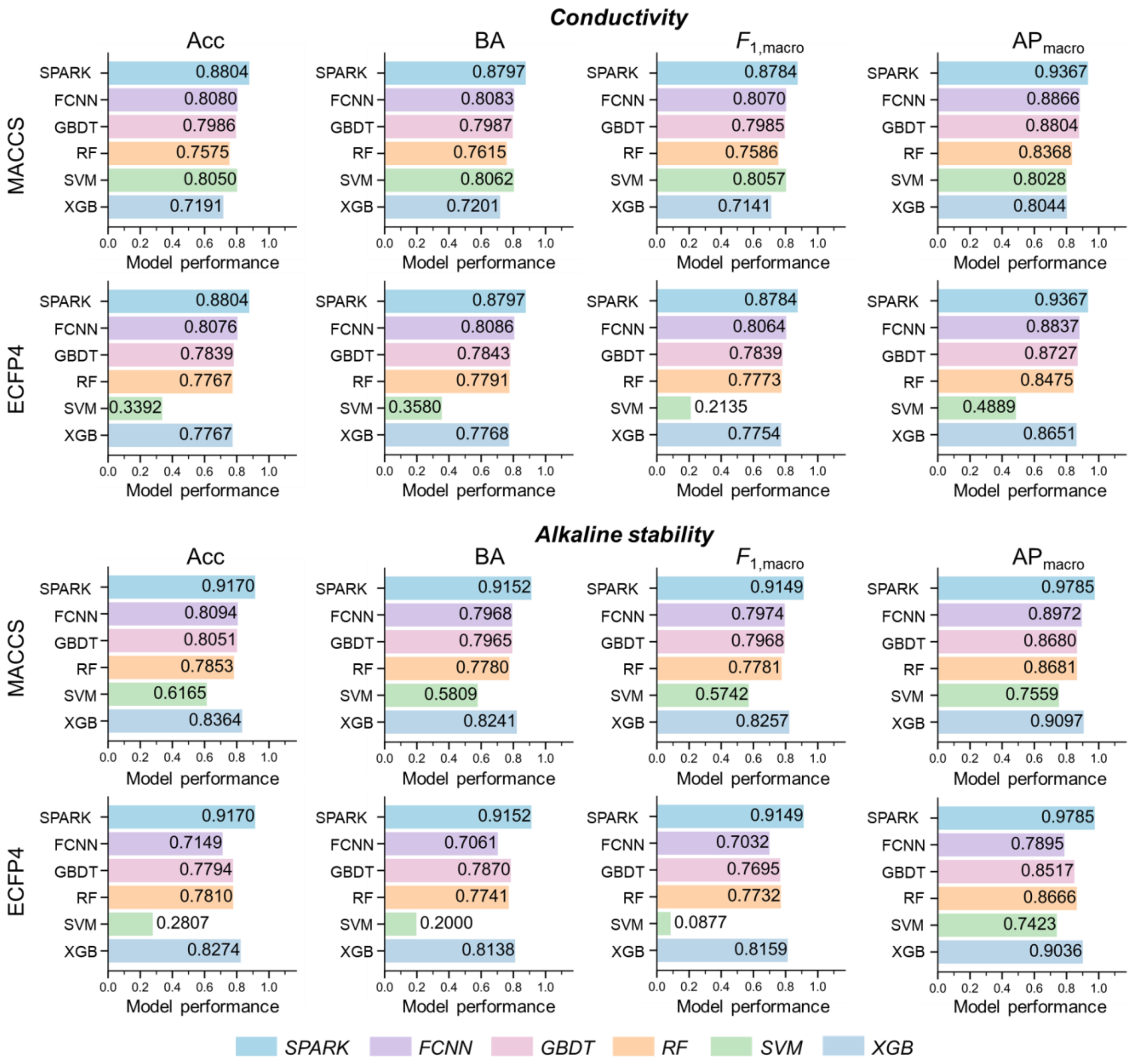

Fig. S8 Detailed performance comparison between SPARK and conventional machine-learning models (FCNN, RF, GBDT, SVR, and XGB trained on ECFP4 and MACCS).

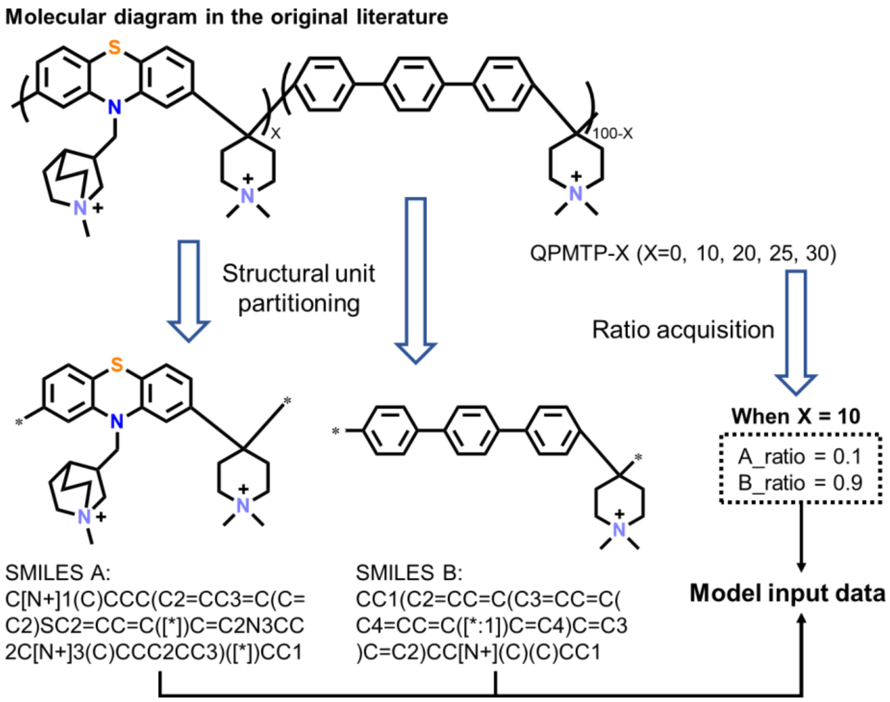

Fig. S9 Schematic workflow for converting AEM structural descriptions from the original literature into the final model input.

Table S1 Detailed information of node (atom) and edge embeddings generated by CHEM module

Types	Feature type	Features	Method	Size	Total size
Nodes	Basic atomic properties	Atomic number	Embedding layer (GROVER)	32	103
		Connectivity	Bucket embedding / numeric processing	15	
		Formal charge	One-hot encoding	3	
		Chirality	One-hot encoding	4	
		Number of attached hydrogens	Numeric representation	1	
		Hybridization type	Bucket embedding	4	
		Aromaticity	One-hot encoding	1	
	Physico-chemical properties	In-ring indicator	One-hot encoding	1	
		Atomic mass	Bucket embedding	8	
		Electronegativity	Bucket embedding	8	
		van der Waals radius	Numeric representation	4	
		Ionization energy / electron affinity	Numeric representation	4	
		Valence electron count	Bucket embedding	8	
	Reactivity	Acidic hydrogen / basic nitrogen	Numeric representation	2	
		Electrophilicity	Numeric representation	4	
		Thioether sulfur	Numeric representation	1	
		Aluminum-bound hydroxyl oxygen	Numeric representation	1	
	Special tags	Pseudo-atom flag	Numeric representation	1	
		Valid-atom flag	Numeric representation	1	
Edges	Basic bond properties	Bond type	Embedding layer (NGG)	12	18
		Stereochemistry	One-hot encoding	4	
		Conjugated bond	Numeric representation	1	
		Ring bond	Numeric representation	1	

Table S2 Hyperparameter settings for the pretraining and fine-tuning

Training Stage	Hyperparameter	Value
Pre-training	Mask ratio	0.3
	Batch size	64
	Learn rate	1e-4
	Weight decay	1e-5
Fine-tuning (Conductivity grading task)	Train epoch	500
	Batch size	8
	Learn rate	1e-3
	Dropout	0.2
	Hidden dims classifier	[256, 128]
	Layers of GAT	3
	Hidden Layer Dimension of GAT	128
Fine-tuning (Stability grading task)	Train epoch	500
	Batch size	128
	Learn rate	1e-3
	Dropout	0.1
	Hidden dims classifier	[256, 128]
	Layers of GAT	4
	Hidden layer dimension of GAT	128

Table S3 Representative SA scores and Tanimoto coefficients for selected structures as illustrative examples.

SMILES of generated AEM repeating units	Max Tanimoto	SA score
*Oc1ccc(C(=O)c2ccc(Oc3c(C)cc(C(C)(C)c4cc(C)c(*)c(C)c4)cc3C[N+](C)(C)CCCCCCC[N+](C)(C)C)cc2)cc1	0.9824	3.8816
*Oc1c(C)cc(C(c2ccc(C[N+](C)(C)C)cc2)c2cc(C)c(Oc3ccc(S(=O)(=O)c4ccc(Oc5ccc(*)cc5)cc4)cc3)c(C)c2)cc1C	0.9804	4.0849
Oc1ccc(C(=O)c2ccc(Oc3ccc(O)c(C[N+](C)(C)CCCCCC[N+](C)(C)CCC[N+](C)(C)C)c3)cc2)cc1	0.9796	3.7568
Oc1ccc(C(=O)c2ccc(Oc3ccc(O)c(C[N+](C)(C)CCCCCCCCC[N+](C)(C)CCC[N+](C)(C)C)c3)cc2)cc1	0.9796	3.7763
Oc1ccc(Oc2ccc(C(=O)c3ccc(Oc4ccc(O)c(C[N+](C)(C)CCC[N+](C)(C)CCCCCCCCC[N+](C)(C)C)c4)cc3)cc2)cc1	0.9796	3.7493
CCCC(C)CC(CC)c1ccc(C[N+](C)(C)CCCCCCC[N+](C)(C)CCCCC[N+](C)(C)C)cc1	0.9778	4.6653
CCCC(C)CC(CC)c1ccc(C[N+](C)(C)CCCCCCC[N+](C)(C)CCCCCCC[N+](C)(C)CCCCC[N+](C)(C)C)cc1	0.9778	4.8147

Table S4 Performance comparison using different pretraining datasets.

Pretraining dataset	Pretraining loss		BA_{test}
	Hydrophilic channel	Hydrophobic channel	
Literature-only data	0.09764	0.15510	0.7857
Generated data only	0.03080	0.04837	0.8676
Literature and generated data	0.02527	0.04342	0.8797

Table S5 Mean (avg), maximum (max), minimum (min), standard deviation (std), and standard error (sem) of the 10-fold training results at the optimal epoch for PT-SPARK and NPT-SPARK.

Learn indicators	Conductivity grading task		Stability grading task	
	PT-SPARK	NPT-SPARK	PT-SPARK	NPT-SPARK
train_loss_avg	0.1539	0.5713	0.1286	0.1961
train_loss_max	0.1626	0.6056	0.1355	0.2032
train_loss_min	0.1418	0.5398	0.1241	0.1854
train_loss_std	0.0061	0.0247	0.0032	0.0050
train_loss_sem	0.0019	0.0078	0.0010	0.0016
val_loss_avg	0.4623	0.9455	0.2098	0.5421
val_loss_max	0.5036	1.0829	0.2280	0.5802
val_loss_min	0.4047	0.7229	0.1861	0.5063
val_loss_std	0.0319	0.0921	0.0108	0.0215
val_loss_sem	0.0101	0.0291	0.0034	0.0068
test_loss_avg	0.4658	0.9397	0.2078	0.5223
test_loss_max	0.5381	1.0816	0.2204	0.6141
test_loss_min	0.4101	0.8075	0.2040	0.4761
test_loss_std	0.0394	0.0865	0.0051	0.0430
test_loss_sem	0.0125	0.0274	0.0016	0.0136
train_acc_avg	0.9427	0.7635	0.9398	0.9147
train_acc_max	0.9470	0.7756	0.9423	0.9189
train_acc_min	0.9395	0.7493	0.9343	0.9103
train_acc_std	0.0023	0.0104	0.0022	0.0024
train_acc_sem	0.0007	0.0033	0.0007	0.0008
val_acc_avg	0.8810	0.6461	0.9201	0.8295
val_acc_max	0.8900	0.6875	0.9257	0.8407
val_acc_min	0.8716	0.6312	0.9171	0.8171
val_acc_std	0.0063	0.0174	0.0034	0.0071
val_acc_sem	0.0020	0.0055	0.0011	0.0023
test_acc_avg	0.8805	0.6458	0.9170	0.8380
test_acc_max	0.8931	0.6782	0.9227	0.8497
test_acc_min	0.8633	0.6165	0.9085	0.8205
test_acc_std	0.0083	0.0236	0.0045	0.0104
test_acc_sem	0.0026	0.0075	0.0014	0.0033

Table S6 Performance comparison of PT-SPARK with NPT-SPARK

		Conductivity grading task		Stability grading task	
		PT-SPARK	NPT-SPARK	PT-SPARK	NPT-SPARK
Training set	Acc	0.9398	0.7634	0.9426	0.9147
	BA	0.9378	0.7581	0.9422	0.9117
	$F_{1,\mathrm{macro}}$	0.9398	0.7575	0.9418	0.9120
	$\mathrm{AP}_{\mathrm{macro}}$	0.9899	0.7587	0.9859	0.9127
Validation set	Acc	0.8810	0.6461	0.9201	0.8295
	BA	0.8807	0.6368	0.9184	0.8257
	$F_{1,\mathrm{macro}}$	0.8796	0.6352	0.9177	0.8240
	$\mathrm{AP}_{\mathrm{macro}}$	0.9384	0.6437	0.9786	0.8244
Test set	Acc	0.8805	0.6455	0.9170	0.8380
	BA	0.8797	0.6357	0.9152	0.8345
	$F_{1,\mathrm{macro}}$	0.8784	0.6347	0.9149	0.8330
	$\mathrm{AP}_{\mathrm{macro}}$	0.9367	0.6431	0.9785	0.8333

Table S7 Test set performance comparison of SPARK with the conventional models

	Models	Acc	BA	$F_{1,\text{macro}}$	AP_{macro}
Conductivity grading task	***SPARK***	***0.8804***	***0.8797***	***0.8784***	***0.9367***
	FCNN-MACCS	0.8080	0.8083	0.8070	0.8866
	GBDT-MACCS	0.7986	0.7987	0.7985	0.8804
	RF-MACCS	0.7575	0.7615	0.7586	0.8368
	SVM-MACCS	0.8050	0.8062	0.8057	0.8028
	XGB-MACCS	0.7191	0.7201	0.7141	0.8044
	FCNN-ECFP4	0.8076	0.8086	0.8064	0.8837
	GBDT-ECFP4	0.7839	0.7843	0.7839	0.8727
	RF-ECFP4	0.7767	0.7791	0.7773	0.8475
	SVM-ECFP4	0.3392	0.3580	0.2135	0.4889
	XGB-ECFP4	0.7767	0.7768	0.7754	0.8651
Stability grading task	***SPARK***	***0.9170***	***0.9152***	***0.9149***	***0.9785***
	FCNN-MACCS	0.8094	0.7968	0.7974	0.8972
	GBDT-MACCS	0.8051	0.7965	0.7968	0.8680
	RF-MACCS	0.7853	0.7780	0.7781	0.8681
	SVM-MACCS	0.6165	0.5809	0.5742	0.7559
	XGB-MACCS	0.8364	0.8241	0.8257	0.9097
	FCNN-ECFP4	0.7149	0.7061	0.7032	0.7895
	GBDT-ECFP4	0.7794	0.7870	0.7695	0.8517
	RF-ECFP4	0.7810	0.7741	0.7732	0.8666
	SVM-ECFP4	0.2807	0.2000	0.0877	0.7423
	XGB-ECFP4	0.8274	0.8138	0.8159	0.9036

Table S8 Training set performance comparison of SPARK with the conventional models

	Models	Acc	BA	$F_{1,macro}$	AP_{macro}
Conductivity grading task	***SPARK***	***0.9426***	***0.9422***	***0.9418***	***0.9859***
	FCNN-MACCS	0.8574	0.8551	0.8558	0.9428
	GBDT-MACCS	0.8567	0.8547	0.8552	0.9363
	RF-MACCS	0.8335	0.8323	0.8318	0.9207
	SVM-MACCS	0.8797	0.8799	0.8792	0.8971
	XGB-MACCS	0.7519	0.7456	0.7438	0.8382
	FCNN-ECFP4	0.8506	0.8512	0.8509	0.9397
	GBDT-ECFP4	0.8579	0.8558	0.8563	0.9382
	RF-ECFP4	0.8615	0.8599	0.8600	0.9455
	SVM-ECFP4	0.3285	0.3614	0.2096	0.4834
	XGB-ECFP4	0.8271	0.8225	0.8236	0.9142
Stability grading task	***SPARK***	***0.9398***	***0.9378***	***0.9398***	***0.9899***
	FCNN-MACCS	0.8319	0.8252	0.8258	0.9181
	GBDT-MACCS	0.9091	0.9053	0.9061	0.9787
	RF-MACCS	0.8295	0.8257	0.8249	0.9181
	SVM-MACCS	0.6686	0.6361	0.6316	0.8476
	XGB-MACCS	0.8734	0.8646	0.8672	0.9502
	FCNN-ECFP4	0.7327	0.7166	0.7164	0.8229
	GBDT-ECFP4	0.9064	0.9137	0.9036	0.9736
	RF-ECFP4	0.8305	0.8273	0.8261	0.9195
	SVM-ECFP4	0.2668	0.2008	0.0857	0.7706
	XGB-ECFP4	0.8626	0.8522	0.8549	0.9405

Table S9 Validation set performance comparison of SPARK with the conventional models

	Models	Acc	BA	$F_{1,\text{macro}}$	AP_{macro}
Conductivity grading task	***SPARK***	***0.8810***	***0.8807***	***0.8796***	***0.9384***
	FCNN-MACCS	0.8019	0.8052	0.8022	0.8832
	GBDT-MACCS	0.7951	0.7974	0.7962	0.8729
	RF-MACCS	0.7616	0.7653	0.7635	0.8330
	SVM-MACCS	0.8030	0.8065	0.8052	0.7987
	XGB-MACCS	0.7134	0.7173	0.7107	0.8052
	FCNN-ECFP4	0.8102	0.8128	0.8099	0.8894
	GBDT-ECFP4	0.7959	0.7984	0.7967	0.8716
	RF-ECFP4	0.7729	0.7762	0.7744	0.8422
	SVM-ECFP4	0.3348	0.3621	0.2148	0.4765
	XGB-ECFP4	0.7755	0.7784	0.7753	0.8666
Stability grading task	***SPARK***	***0.9201***	***0.9184***	***0.9177***	***0.9786***
	FCNN-MACCS	0.7930	0.7799	0.7822	0.8846
	GBDT-MACCS	0.7913	0.7826	0.7833	0.8614
	RF-MACCS	0.7673	0.7591	0.7595	0.8559
	SVM-MACCS	0.6457	0.6168	0.5972	0.8207
	XGB-MACCS	0.8115	0.7988	0.8004	0.8984
	FCNN-ECFP4	0.7007	0.6911	0.6902	0.7854
	GBDT-ECFP4	0.8116	0.8069	0.7984	0.8759
	RF-ECFP4	0.7638	0.7564	0.7559	0.7559
	SVM-ECFP4	0.2814	0.2000	0.0878	0.7871
	XGB-ECFP4	0.8042	0.7906	0.7924	0.8889

Table S10 Results of the temperature-boundary test

	Low-temperature data (T< 80°C)			Unseen high-temperature data (T ≥ 80°C)
	Training set	Validation set	Test set	External test set
Balanced accuracy	0.9517	0.8952	0.8863	0.7027

Table S11 Results for model ablation experiments based on test set

	Models	Acc	BA	$F_{1,\text{ macro}}$	AP_{macro}
Conductivity grading task	***SPARK***	***0.8804***	***0.8797***	***0.8784***	***0.9367***
	GAT[a]	0.5696	0.5668	0.5505	0.6105
	E-CHEM[b]	0.8068	0.8057	0.8048	0.8942
	E-DcP[c]	0.8200	0.8198	0.8177	0.8951
	N-FiLM[d]	0.8415	0.8417	0.8396	0.9165
Stability grading task	***SPARK***	***0.9170***	***0.9152***	***0.9149***	***0.9785***
	GAT[a]	0.7510	0.7387	0.7382	0.8306
	E-CHEM[b]	0.8300	0.8190	0.8193	0.8832
	E-DcP[c]	0.8136	0.8020	0.8021	0.8711
	E-FiLM[d]	0.8815	0.8739	0.8759	0.9624

[a]Simultaneous removal of all three feature modules (CHEM, DcP, and FiLM), retaining only the base graph encoder (GAT); [b]Removal of CHEM module; [c]Removal of the dual-channel pretraining architecture (DcP); [d]Removal of FiLM.

Table S12 Training set results for model ablation experiments

	Models	Acc	BA	$F_{1,\text{macro}}$	AP_{macro}
Conductivity grading task	***SPARK***	***0.9426***	***0.9422***	***0.9418***	***0.9859***
	GAT[a]	0.6188	0.6133	0.5958	0.6722
	E-CHEM[b]	0.8850	0.8850	0.8836	0.9568
	E-DcP[c]	0.8461	0.8458	0.8445	0.9224
	N-FiLM[d]	0.8707	0.8702	0.8692	0.9477
Stability grading task	***SPARK***	***0.9398***	***0.9378***	***0.9398***	***0.9899***
	GAT[a]	0.7586	0.7432	0.7443	0.8397
	E-CHEM[b]	0.8392	0.8279	0.8293	0.8955
	E-DcP[c]	0.8370	0.8259	0.8272	0.8930
	N-FiLM[d]	0.8980	0.8932	0.8943	0.9651

[a]Simultaneous removal of all three feature modules (CHEM, DcP, and FiLM), retaining only the base graph encoder (GAT); [b]Removal of CHEM module; [c]Removal of the dual-channel pretraining architecture (DcP); [d]Removal of FiLM.

Table S13 Validation set results for model ablation experiments

	Models	Acc	BA	$F_{1,\text{ macro}}$	AP_{macro}
Conductivity grading task	***SPARK***	***0.8810***	***0.8807***	***0.8796***	***0.9384***
	GAT[a]	0.5812	0.5725	0.5559	0.6164
	E-CHEM[b]	0.8079	0.8043	0.8048	0.8856
	E-DcP[c]	0.8139	0.8130	0.8106	0.8935
	N-FiLM[d]	0.8557	0.8549	0.8530	0.9212
Stability grading task	***SPARK***	***0.9201***	***0.9184***	***0.9177***	***0.9786***
	GAT[a]	0.7389	0.7216	0.7207	0.8189
	E-CHEM[b]	0.8184	0.8043	0.8046	0.8625
	E-DcP[c]	0.8128	0.7982	0.7974	0.8470
	N-FiLM[d]	0.8785	0.8717	0.8733	0.9506

[a]Simultaneous removal of all three feature modules (CHEM, DcP, and FiLM), retaining only the base graph encoder (GAT); [b]Removal of CHEM module; [c]Removal of the dual-channel pretraining architecture (DcP); [d]Removal of FiLM.

Table S14 Detailed information of node (atom) and edge (bond) embeddings generated by RDKit in the E-CHEM experiments

	Features	Total size
Nodes	Atomic number<br>connectivity<br>Formal charge<br>Chirality<br>Number of attached hydrogens<br>Hybridization type<br>Aromaticity<br>Is-in-ring<br>Total valence<br>Implicit valence<br>Number of radical electrons	11
Edges	Single bond<br>Double bond<br>Triple bond<br>Aromatic bond<br>Conjugation<br>Is-in-ring	6

Table S15 Results of the scaffold-split validation experiment.

	Seen-scaffold data			Unseen-scaffold data
	Training set	Validation set	Test set	External test set
Balance accuracy	0.9422	0.8681	0.8734	0.8021

Table S16 Comparison between experimentally measured grades and model-predicted grades.

Task	Sample	True value	$Level_{True}$	$Level_{Pre}$	Ref.
Conductivity grading task	P1NTP	155 mS/cm[a]	1	1	[43]
	P2TPN	115 mS/cm[a]	3	3	[43]
	qPTOCB	155 mS/cm[a]	1	1	[44]
	QTPTF-OPF	152.5 mS/cm[a]	1	1	[45]
Stability grading task	P1NTP	95.2%[b]	1	1	[43]
	P2TPN	88.9%[b]	2	1	[43]
	PTOCB	90.29%[c]	1	1	[44]
	QTPTF-OPF	91.03%[d]	1	1	[45]

[a]Measured at 80 °C; [b]Conductivity retention after immersion in 80 °C 1 M alkaline solution for 3000 h; [c]Conductivity retention after immersion in 80 °C 2 M alkaline solution for 1000 h; [d]Conductivity retention after immersion in 80 °C 3 M alkaline solution for 500 h.

Table S17 Definitions of per-grade performance evaluation metrics

Metrics	Definitions
Precision_i	The proportion of correctly predicted positive samples among all samples predicted as positive.
Balanced accuracy (BA_i)	The arithmetic mean of recall across all classes.
Recall_i	The proportion of true positive samples that are correctly identified, reflecting the model's sensitivity to the positive class.
F1-Score ($F_{1,i}$)	The harmonic mean of precision and recall.
Specificity_i	The proportion of true negative samples that are correctly identified, reflecting the model's ability to recognize the negative class.
Negative predictive value (NPV_i)	The proportion of correctly predicted negative samples among all samples predicted as negative, indicating the reliability of negative predictions.
False positive rate (FPR_i)	The proportion of negative samples incorrectly classified as positive, indicating the tendency toward false alarms.
False negative rate (FNR_i)	The proportion of positive samples incorrectly classified as negative, indicating the tendency to miss positive cases.
Receiver operating characteristic curve (ROC_i)	The classification performance across different decision thresholds.
Area under the ROC curve (AUC_i)	The model's ability to discriminate between positive and negative classes.

Table S18 Definitions of overall performance evaluation metrics for multi-class classification tasks

Metrics	Definitions
Accuracy (Acc)	The proportion of correctly classified samples among all samples
Balanced accuracy (BA)	The arithmetic mean of recall scores across all classes
Macro-averaged F1-score ($F_{1,\mathrm{macro}}$)	A harmonic measure balancing precision and recall across all classes
Macro-average Precision (P_{macro})	The arithmetic mean of precision scores across all classes.
Macro-average Recall (R_{macro})	The arithmetic mean of recall scores across all classes.
Micro-average F1-score ($F_{1,\mathrm{micro}}$)	The F1-score calculated by aggregating total true positives, false negatives, and false positives globally.
Micro-average Precision (P_{micro})	The global recall calculated by summing the true positives and false negatives across all classes.
Micro-average Recall (R_{micro})	Calculated from globally aggregated TPs and FNs.
Cohen's Kappa coefficient (κ)	The statistic that measures inter-rater agreement for categorical items, correcting for agreement occurring by chance.
Macro-average AUC-ROC ($\mathrm{AR_{macro}}$)	The arithmetic mean of AUC scores across all classes
